# On an SE(Is)(Ih)AR epidemic model with combined vaccination and antiviral controls for COVID-19 pandemic

**DOI:** 10.1186/s13662-021-03248-5

**Published:** 2021-02-01

**Authors:** M. De la Sen, A. Ibeas

**Affiliations:** 1 Institute of Research and Development of Processes IIDP, Leioa, Spain; 2 Department of Telecommunications and Systems Engineering, Universitat Autònoma de Barcelona, UAB, 08193 Barcelona, Spain

**Keywords:** SEIR epidemic model, SE(Is)(Ih)AR epidemic model, Vaccination control, Antiviral treatment control, Reproduction number, Nonnegativity of solutions, Limit cycles

## Abstract

In this paper, we study the nonnegativity and stability properties of the solutions of a newly proposed extended SEIR epidemic model, the so-called SE(Is)(Ih)AR epidemic model which might be of potential interest in the characterization and control of the COVID-19 pandemic evolution. The proposed model incorporates both asymptomatic infectious and hospitalized infectious subpopulations to the standard infectious subpopulation of the classical SEIR model. In parallel, it also incorporates feedback vaccination and antiviral treatment controls. The exposed subpopulation has three different transitions to the three kinds of infectious subpopulations under eventually different proportionality parameters. The existence of a unique disease-free equilibrium point and a unique endemic one is proved together with the calculation of their explicit components. Their local asymptotic stability properties and the attainability of the endemic equilibrium point are investigated based on the next generation matrix properties, the value of the basic reproduction number, and nonnegativity properties of the solution and its equilibrium states. The reproduction numbers in the presence of one or both controls is linked to the control-free reproduction number to emphasize that such a number decreases with the control gains. We also prove that, depending on the value of the basic reproduction number, only one of them is a global asymptotic attractor and that the solution has no limit cycles.

## Introduction

Along the last two decades, an important effort has been devoted to the research of mathematical epidemic models based on integro-differential equations and/or difference equations. Such models describe the evolution through time of various subpopulations integrated in the epidemic model. The classical so-called SEIR (Susceptible–Exposed–Infectious–Recovered) epidemic model splits the infectious population into two subpopulations (or compartments), namely, the so-called “infected” or “exposed” (E) (those having the disease but with no external symptoms) and the “infectious” or “infective” (I) (those having external symptoms). The SEIR model has multiple variants with different degrees of complexity, including those admitting controls like, for instance, constant and feedback vaccination and treatment controls and/or impulsive controls (exerted on very short periods of time) or those involving several interacting patches associated with different towns or regions; see, for instance, [1–3] and [4–12] and references therein. In [11] an epidemic model subject to a ratio-dependent saturation incidence rate is proposed. On the other hand, a new SIR (Susceptible–Infectious–Recovered) epidemic model under impulsive vaccination is investigated in [12], and a nonautonomous SIRVS epidemic model with vaccination controls is proposed and studied in [13]. Also, an epidemic delayed model with diffusion is characterized and studied in [14]. It is worth mentioning that a relevant attention has been paid to the investigation of the stability and nonnegativity and positivity properties of epidemic models in both vaccination-free and vaccination control situations. See, for instance, [14–26], and also [17, 18] in the stochastic framework context. On the other hand, we can point out that the nonnegativity of the solution is commonly required in biological processes to appropriately approach in a coherent fashion their natural evolution. See, for instance, [27] and some references therein, concerning the Beverton–Holt equation of population model evolution at successive stages.

On the other hand, it is known that there may be some individuals who are infective but have no significant external symptoms, the so-called “asymptomatic” (A) subpopulation; see [28–32], and references therein. This occurs even in the common known influenza disease. If such an asymptomatic subpopulation is incorporated to the model, then it turns out that the exposed have different transitions to the infective and to the asymptomatic in such a way that a proportion of the exposed become asymptomatic after a certain time period while others become infectious. Note, for instance, that in the Ebola disease the lying dead corpses are also infective [4, 28–31, 33], which can cause very serious sanitary problems in third-world tropical countries with low or scarce sanitary means. In particular, SEIADR-type epidemic models are considered in [29–31], which incorporate asymptomatic and dead populations to the typical SEIR models and which include, in general, vaccination and treatment controls as well as impulsive controls to retire the infective bodies from the streets in third-world countries hit by Ebola outbreaks.

In this paper, we propose and investigate an extended SEIR model with six subpopulations, the so-called SE(Is)(Ih)AR. There are four infective subpopulations integrated in such a model, which are the exposed subpopulation, the symptomatic slight infectious subpopulation, the symptomatic serious infection subpopulation, and the asymptomatic subpopulation. It is important to specifically consider the seriously infectious subpopulation as a separate one from the slightly infectious individuals due to their high consumption of hospital resources (intensive care attention means, respirators, etc.) and special staff attention related to the slight infectious individuals. Each individual of exposed subpopulation has a transition either to one of the symptomatic infectious subpopulation or to the asymptomatic one. The respective transmission rates are different in general because of different reasons; for instance, the asymptomatic individuals do not cough or can cough occasionally due to other reasons than COVID disease such as allergies, asthma, or gastroesophageal reflux disease, so it is expected that their transmission rate for contagion of susceptible individuals is smaller than that of the infectious ones. Also, the hospitalized individuals do not contact usually the same average numbers of susceptible as the asymptomatic or slight infectious contact, whereas the hospital staff members that contact with them are expected to have protection suits and sanitary contact means. So, it is also expected that the hospitalized individuals have a smaller transmission rates than those of the slight infectious ones. The proposed epidemic model is also subject, in the most general framework, to feedback vaccination and treatment controls. It is tested through numerical worked and tested examples under parameterizations related to the recent COVID-19 pandemic, which is exhaustively studied in the medical and computational background literature; see, for instance, [34–48] and references therein. Therefore the current study is of important potential interest, although nowadays there is no yet an approved vaccine for COVID-19 to be applied on the population. It turns out that COVID-19 is a respiratory viral infectious disease, which has a very high contagiousness related to the typical influenza or the known common cold. Therefore intervention rules, such as confinements, social distance keeping, limitation of events, and attendance numbers or use of face masks, are recommended or even mandatory to mitigate the disease spread. The use of masks is probably the simpler, cheaper, and most efficient weapon against the pandemic, although it can have also secondary effects on the health [43], mainly due to the incomplete ejection of carbon dioxide outside with the exhalation of air phase. COVID-19 pandemic also exhibits very different symptoms and later secondary effects depending on the particular infected individual running from asymptomatic, or very slight, symptoms to very serious ones, needing extreme hospital care, sometimes producing serious damage in organs like lungs, liver, or heart and sometimes ending with the patient’s death. In particular, the particle swarm optimization algorithm (PSO) is used to estimate an SEIR model parameterization of COVID-19 using available Hubei province data. Also, a fractional-order model SEIRD model (an SEIR model, which includes deceased) is proposed in [35] for COVID-19 pandemic emphasizing that the fractional models possess an inherent memory effect. On the other hand, an epidemic model for COVID-19 that takes into account undetected infective cases and different sanitary and infectiousness conditions of the hospitalized individuals is discussed in [36], whereas an extended SEIR model is considered in [37], which incorporates as a new subpopulation the concentration of the coronavirus in the environment reservoir. Also, the dynamics of such a concentration is driven by the exposed and infectious subpopulations. Ageing population layers for control interventions and re-susceptibility and time delay are considered in [38].

Some new recent work [44] is devoted to discussion of the relevance of lockdowns and quarantines to fight against the spread of the recent COVID-19 pandemic. Further work in the fractional framework for a SIRV model under combined vaccination and treatment controls is reported in [45] and also in [49] concerning rubella propagation. On the other hand, the use of vaccination controls for an SEIRS model under temporary immunity is discussed in [50], whereas the use of impulsive vaccination under short term immunity is proposed in [51] for an SEIR epidemic model. Also, polynomial approaches for the analysis of epidemic models were also proposed. In particular, the use of a Hermite polynomial approach for the solution of an SIR epidemic model is discussed in [52].

The paper is organized as follows. In Sect. 2, we establish a new SE(Is)(Ih)AR epidemic model, which involves six subpopulations, namely, Susceptible (S), Exposed (E), Slight Symptomatic Infectious $I_{s}$ (not requiring hospital care), Seriously Symptomatic Infectious, or hospitalized, $I_{h}$ (requiring hospital care), Asymptomatic (A), and Recovered (R). The exposed subpopulation has transitions to the slight, hospitalized, and asymptomatic infectious, in general, under distinct proportions, and those proportions belong to the set of parameters of the model as it has been previously mentioned. In general, one defines a basic transmission rate for contacts of slight infectious to susceptible, whereas the other two transmission rates for asymptomatic versus susceptible and hospitalized versus susceptible are characterized by relative transmission rates related to the above basic one. On the other hand, it is assumed that the disease mortality affects the fraction of the hospitalized subpopulation only. The model is subject eventually to two different feedback controls, which can be combined, namely, the vaccination control on the susceptible and the treatment control on the hospitalized infectious. In general, the transmission rate and the feedback control gains can be time-varying. The property of nonnegativity of any solution under any nonnegative initial conditions is investigated and proved as well as the boundedness of all the subpopulations for all time, which is a global Lyapunov stability property. Section 3 is devoted to the characterization of the location of the disease-free and the endemic equilibrium points, which are proved to be unique, and to proving their local asymptotic stability conditions. It is seen that the disease-free equilibrium point is locally asymptotically stable when the basic reproduction number is smaller than unity. It is proved that the endemic equilibrium point is attainable (or reachable) in the sense that the nonnegativity of the solutions is kept for all time, as the disease-free equilibrium point is unstable. On the other hand, it is also emphasized that the equilibrium points are dependent on the basic reproduction number (and, equivalently, on the transmission rate if all the remaining model parameters are fixed) and that increase of the values of control gains reduces the value of the basic reproduction number. As a result, the disease-free equilibrium point can be an attractor for higher values of the transmission rate in comparison with the control-free case. It is also shown that no limit cycle can surround any or both equilibrium points if the transmission rate and the control gains converge asymptotically to constant values. As a result, no limit cycle exists under weak conditions on the parameterization of the uncontrolled model and the control gains, whereas only one of the two equilibrium points is a global asymptotic attractor depending on the current value of the basic reproduction number compared to unity.

Section 4 is devoted to the discussion of some numerical examples based on previously tested parameterizations of COVID-19, which are available in the background literature. Finally, some conclusions end the paper.

## The SE(Is)(Ih)AR epidemic model

The proposed SE(Is)(Ih)AR model is an extended SEIR model with the following characteristics: It includes the subpopulations “Susceptible” (S), “Exposed”, who are infected but not yet infective (E), “Slight Symptomatic Infective” or “Slight Symptomatic Infectious” ($I_{s}$), “Seriously Symptomatic Infectious” or “Hospitalized”$( I_{h} )$, “Asymptomatic Infectious” (A), and “Recovered” (R). These subpopulations are appropriate to describe COVID-19, where there is a wide range of influence of the virus on different people, and the model may be the basis of a generic classification of the infectious population into asymptomatic individuals, slightly infectious individuals, and hospitalized individuals. The tested slightly infectious individuals and the asymptomatic ones stay typically at home or in ad hoc prepared and monitored lodgings until further recovery. The slight infectious, serious infectious, and asymptomatic individuals are considered distinct subpopulations since they are originated by different transitions from the exposed subpopulation. Furthermore, the slight and asymptomatic individuals do not need hospital treatment.

The proposed model also incorporates two optional feedback control actions, the standard vaccination control $V ( t )$ on the susceptible and the antiviral treatment $T ( t )$ on the hospitalized infectious subpopulation. The slight and asymptomatic infectious do not need intensive treatment. Therefore the vaccination control is applied to the susceptible individuals, and the treatment control is applied to the hospitalized or seriously infectious individuals. Through the paper, we both formally and intuitively emphasize how those controls help to reduce the reproduction number. See, for instance, [29–31] and [36–38] and references therein for the motivation and use of the controls and the modeling issues for COVID-19, respectively. The SE(Is)(Ih)AR model to be discussed is the following one:

1 $$\begin{aligned} &\dot{S} ( t ) = b_{1} - \bigl[ b_{2} + \beta ( t ) \bigl( I_{s} ( t ) + \beta _{hr}I_{h} ( t ) + \beta _{ar}A ( t ) \bigr) + k_{V} ( t ) \bigr] S ( t ) + \eta R ( t ), \end{aligned}$$

2 $$\begin{aligned} &\dot{E} ( t ) = - ( b_{2} + \gamma ) E ( t ) + \beta ( t ) \bigl( I_{s} ( t ) + \beta _{hr}I_{h} ( t ) + \beta _{ar}A ( t ) \bigr) S ( t ), \end{aligned}$$

3 $$\begin{aligned} &\dot{I}_{s} ( t ) = - ( b_{2} + \tau _{0} ) I_{s} ( t ) + \gamma p_{s} E ( t ), \end{aligned}$$

4 $$\begin{aligned} &\dot{I}_{h} ( t ) = - \bigl( b_{2} + \alpha + \tau _{0} + k_{T} ( t ) \bigr) I_{h} ( t ) + \gamma p_{h} E ( t ), \end{aligned}$$

5 $$\begin{aligned} &\dot{A} ( t ) = - ( b_{2} + \tau _{0} ) A ( t ) + \gamma ( 1 - p_{s} - p_{h} ) E ( t ), \end{aligned}$$

6 $$\begin{aligned} &\dot{R} ( t ) = - ( b_{2} + \eta ) R ( t ) + \tau _{0} \bigl( I_{s} ( t ) + I_{h} ( t ) + A ( t ) \bigr) + k_{T} ( t ) I_{h} ( t ) + k_{V}S ( t ), \end{aligned}$$

for $t\geq 0$ with initial conditions $S ( 0 ) = S_{0}$, $E ( 0 ) = E_{0}$, $I_{s} ( 0 ) = I_{s0}$, $I_{h} ( 0 ) = I_{h0}$, $A ( 0 ) = A_{0}$, and $R ( 0 ) = R_{0}$ subject to $\min ( S_{0}, E_{0}, I_{s0}, I_{h0}, A_{0}, R_{0} ) \ge 0$, where: $b_{1}$ is the recruitment rate, $b_{2}$ is the natural average death rate, $\beta ( t ), \beta _{hr}\beta ( t ), \beta _{ar}\beta ( t )$ are the transmission rates to the susceptible from the respective slight (un-hospitalized) symptomatic infectious, serious (hospitalized) symptomatic infectious, and asymptomatic infectious subpopulations, *η* is a parameter such that $1/\eta $ is the average duration of the immunity period reflecting a transition from the recovered to the susceptible, *γ* is the transition rate from the exposed to all (i.e. both symptomatic and asymptomatic) infectious, *α* is the average extra mortality associated with the symptomatic infectious subpopulation, $\tau _{0}$ is the natural immune response rate for the whole infectious subpopulation (i.e. $A + I$), $p_{s}, p_{h}, p_{a} = 1 - p_{s} - p_{h}$ are the fractions of the exposed that become slight symptomatic infectious, serious symptomatic infectious, and asymptomatic infectious, respectively.

$V ( t ) = k_{V} ( t )S ( t )$ and $T ( t ) = k_{T} ( t )I_{h} ( t )$ are, respectively, the vaccination and antiviral treatment linear feedback controls on the susceptible and hospitalized infectious, respectively, of gains $k_{V}, k_{T}:\mathbf{R}_{0 +} \to \mathbf{R}_{0 +} $.

Note that deterministic models offer a balanced trade-off between complexity and reality representation capabilities. In this way, they are able to reproduce accurately the real behavior of the spreading while maintaining a lower degree of mathematical complexity that allows gaining deep insight into the underlying aspects of the propagation. On the other hand, it has been recently reported that sometimes simple models can give good results in the research on COVID-19. See, for instance, [53], where the main objective is determining the rates of infective contacts along different periods of time. Therefore, in this study, we prefer a deterministic framework for the model in contrast to more sophisticated ones. On the other hand, the integrated inclusion of four infective subpopulations, namely, exposed, asymptomatic, slight asymptomatic infectious, and seriously infectious requiring hospital care, with three different transitions from the exposed individuals to various subpopulation of infectious, is well adapted to the transmission characteristics of the recent COVID-19 pandemic.

### Remark 1

In the above parameterization, all parameters are positive while we assume that $\beta ( t )$ can be time-varying in general. This is a reasonable assumption due to different factors like seasonality or geographic area of application (for instance, rural, populated or with very high population density), which can influence the contacts infectious—susceptible, or the public intervention actions (like confinement, quarantines, or isolation measures), which also modify the average number of infective contagions. The relative values of the other two transmission rates $\beta _{hr}$ and $\beta _{ar}$ are assumed to be constant. In practice, the primary infectivity of the hospitalized infectious can be smaller than that of the slight ones $\beta ( t )$ due to potentially taken protection measures on the hospital staff related and due to the fact that they have less numbers of contacts than average. The transmission rates of the asymptomatic infectious can also be smaller than $\beta ( t )$ due, for instance, to the fact that they cough less intensively. So, it will not be surprising that the values of the relative transmission rates from the hospitalized and asymptomatic $\beta _{hr}$ and $\beta _{ar}$ to the susceptible might typically be less than one.

The following result relies on the solutions in closed form of the proposed SE(Is)(Ih)AR epidemic model, which will be also used to prove the nonnegativity of any solution under any given arbitrary nonnegative initial conditions.

### Theorem 1

*Each solution of the SE*(*Is*)(*Ih*)*AR model* (1)*–*(6) *is uniquely defined*, *and it is nonnegative all the time for any given nonnegative initial conditions and any given vaccination and antiviral controls* $V ( t ) = k_{V} ( t )S ( t )$ *and* $T ( t ) = k_{T} ( t )I_{h} ( t )$ *of gains* $k_{V}, k_{T}:\mathbf{R}_{0 +} \to \mathbf{R}_{0 +} $. *Each solution is expressed in closed form as follows*:

7 $$\begin{aligned} &S ( t ) = e^{ - \int _{ 0}^{ t} \Phi ( \tau ) \,d\tau } S_{0} + \int _{0}^{t} e ^{ - \int _{ \tau }^{ t} \Phi ( \xi ) \,d\xi } \bigl( b_{1} + \eta R ( \tau ) \bigr) \,d\tau,\quad \forall t \in \mathbf{R}_{0 +}, \end{aligned}$$

8 $$\begin{aligned} &E ( t ) = e^{ - ( b_{2} + \gamma ) t}E_{0} + \int _{ 0}^{ t} e^{ - ( b_{2} + \gamma ) ( t - \tau )}\Psi ( \tau ) S ( \tau )\,d\tau,\quad \forall t \in \mathbf{R}_{0 +}, \end{aligned}$$

*from* (1)*–*(2), *where*

9 $$ \Phi ( t ) = \Psi ( t ) + b_{2} + k_{V} ( t ),\qquad \Psi ( t ) = \beta ( t ) \bigl( I_{s} ( t ) + \beta _{hr}I_{h} ( t ) + \beta _{ar}A ( t ) \bigr), \quad\forall t \in \mathbf{R}_{0 +}. $$

*Also*, *from* (3)*–*(6) *we get that*

10 $$\begin{aligned} &I_{s} ( t ) = e^{ - ( b_{2} + \tau _{0} ) t}I_{s0} + \gamma p_{s} \int _{ 0}^{ t} e^{ - ( b_{2} + \tau _{0} ) ( t - \tau )}E ( \tau )\,d\tau,\quad \forall t \in \mathbf{R}_{0 +}, \end{aligned}$$

11 $$\begin{aligned} &I_{h} ( t ) = e^{ - ( b_{2} + \alpha + \tau _{0} ) t - \int _{0}^{t} k_{T} ( \tau ) \,d\tau } I_{h0} + \gamma p_{h} \int _{ 0}^{ t} e^{ - ( b_{2} + \alpha + \tau _{0} ) ( t - \tau ) - \int _{\tau }^{t} k_{T} ( \xi ) \,d\xi } E ( \tau )\,d\tau, \\ &\quad \forall t \in \mathbf{R}_{0 +}, \end{aligned}$$

12 $$\begin{aligned} &A ( t ) = e^{ - ( b_{2} + \tau _{0} ) t}A ( 0 ) + \gamma ( 1 - p_{s} - p_{h} ) \int _{ 0}^{ t} e^{ ( b_{2} + \tau _{0} ) ( t - \tau )}E ( \tau )\,d\tau, \quad \forall t \in \mathbf{R}_{0 +}, \end{aligned}$$

13 $$\begin{aligned} &R ( t ) = e^{ - ( b_{2} + \eta ) t}R_{0} + \int _{ 0}^{ t} e^{ ( b_{2} + \eta ) \tau } \Omega ( \tau ) \,d \tau , \quad\forall t \in \mathbf{R}_{0 +}, \end{aligned}$$

*where* $\Omega ( t ) = \tau _{0} ( I_{s} ( t ) + I_{h} ( t ) + A ( t ) ) + k_{T} ( t ) I_{h} ( t ) + k_{V}S ( t )$, $\forall t \in \mathbf{R}_{0 +} $. *Now from* (7) *we have that* $S_{0} \ge 0 \Rightarrow S ( t ) \ge 0$, $\forall t \in \mathbf{R}_{0 +} $, *and since* $E_{0} \ge 0$ *and* $S ( t ) \ge 0$, $\forall t \in \mathbf{R}_{0 +} $, *from* (8) *we have* $E ( t ) \ge 0$, $\forall t \in \mathbf{R}_{0 +} $. *Then from* (10)*–*(12) *it follows that* $I_{s} ( t ) \ge 0$, $I_{h} ( t ) \ge 0$, *and* $A ( t ) \ge 0$, $\forall t \in \mathbf{R}_{0 +} $, *since* $E ( t ) \ge 0$, $\forall t \in \mathbf{R}_{0 +} $, *and* $I_{s0} \ge 0, I_{h0} \ge 0$, *and* $A_{0} \ge 0$. *Finally*, *from* (13) *it follows that* $R ( t ) \ge 0$, $\forall t \in \mathbf{R}_{0 +} $, *since* $R_{0} \ge 0$ *and* $\Omega ( t ) \ge 0$, $\forall t \in \mathbf{R}_{0 +} $. *The proof is complete*.

The boundedness of the subpopulations all the time is proved in the subsequent result, whose proof is supported by the nonnegativity of the state-trajectory solution concluded from Theorem 1.

### Theorem 2

*We have the following properties under the assumptions of Theorem* 1:

(i) $\lim \sup_{t \to \infty } I_{h} ( t ) \le b_{1}/\alpha $,
(ii) *The total population* $N ( t ) = S ( t ) + E ( t ) + I_{s} ( t ) + I_{h} ( t ) + A ( t ) + R ( t )$ *is bounded for* $t \in \mathbf{R}_{0 +}$ *under any initial finite conditions*
(iii) $\max ( \sup_{t \in \mathbf{R}_{0 +}} S ( t ), \sup_{t \in \mathbf{R}_{0 +}} E ( t ), \sup_{t \in \mathbf{R}_{0 +}} I_{s} ( t ), \sup_{t \in \mathbf{R}_{0 +}} I_{h} ( t ), \sup_{t \in \mathbf{R}_{0 +}} A ( t ), \sup_{t \in \mathbf{R}_{0 +}} R ( t ) ) < + \infty $ *for any given finite nonnegative initial conditions*. *As a result*, *system* (1)*–*(6) *is globally Lyapunov stable*.

### Proof

Assume that $\lim \sup_{t \to \infty } I ( t ) > b_{1}/\alpha $ and proceed by contradiction. By summing up (1)–(6) we get:

14 $$ \dot{N} ( t ) = - b_{2}N ( t ) + b_{1} - \alpha I_{h} ( t ),\quad \forall t \in \mathbf{R}_{0 +}, $$

which leads to the following unique solution for any given $N ( 0 ) = N_{0}$:

15 $$ N ( t ) = e^{ - b_{2} t} \biggl( N_{0} + \int _{0}^{t} e^{b_{2} \tau } \bigl( b_{1} - \alpha I_{h} ( \tau ) \bigr) \,d\tau \biggr),\quad \forall t \in \mathbf{R}_{0 +}. $$

We proceed by contradiction by assuming that $\lim \sup_{t \to \infty } I_{h} ( t ) > b_{1}/\alpha $. Then there is a finite $t_{f} \in \mathbf{R}_{0 +}$ such that $I_{h} ( t ) > b_{1}/\alpha $, $\forall t \in \Delta = [ t_{f}, \infty )\backslash \Delta _{0}$, where $\Delta _{0} \subset [ t_{f},\infty ) \cap \mathbf{R}_{0 +}$ is empty or nonempty but of zero Lebesgue measure. Note that Δ has infinite Lebesgue measure by construction. Thus from (15) we have:

16 $$\begin{aligned} &\mathop{\lim \inf}_{t \to \infty } \biggl( - N ( t ) + \int _{0}^{t_{f}} e^{ - b_{2} ( t - \tau )} \bigl( b_{1} - \alpha I_{h} ( \tau ) \bigr) \,d\tau - \int _{t_{f}}^{t} e^{ - b_{2} ( t - \tau )} \bigl( \alpha I_{h} ( \tau ) - b_{1} \bigr) \,d\tau \biggr) \\ &\quad= \mathop{\lim \inf}_{t \to \infty } \biggl( - N ( t ) + \int _{0}^{t_{f}} e^{ - b_{2} ( t - \tau )} \bigl( b_{1} - \alpha I_{h} ( \tau ) \bigr) \,d\tau - \int _{\Delta } e^{ - b_{2} ( t - \tau )} \bigl( \alpha I_{h} ( \tau ) - b_{1} \bigr) \,d\tau \biggr) \\ &\quad \ge \mathop{\lim \inf}_{t \to \infty } \biggl( - N ( t ) + C ( t_{f} ) - \frac{1 - e^{ - b_{2} ( t - t_{f} )}}{b_{2}} \int _{\Delta } e^{ - b_{2} ( t - \tau )} \bigl( \alpha I_{h} ( \tau ) - b_{1} \bigr) \,d\tau \biggr) \ge 0, \end{aligned}$$

where $C ( t ) = \int _{0}^{t} e^{ - b_{2} ( t - \tau )} \vert b_{1} - \alpha I_{h} ( \tau ) \vert \,d\tau $, $\forall t \in \mathbf{R}_{0 +} $, implies that $C ( t_{f} ) < + \infty $ since $[ 0, t_{f} )$ is a finite interval and the integrand is a continuous and thus bounded function of time, and $\frac{1 - e^{ - b_{2} t_{f}}}{b_{2}}\int _{\Delta } e^{ - b_{2} ( t - \tau )} ( \alpha I_{h} ( \tau ) - b_{1} ) \,d\tau = + \infty $. Then $\lim_{t \to \infty } N ( t ) = - \infty $, a contradiction if $\lim \sup_{t \to \infty } I_{h} ( t ) \le b_{1}/\alpha $ does not hold. As a result, $\lim \sup_{t \to \infty } I_{h} ( t ) \le b_{1}/\alpha $, and property (i) is proved. Now, from property (i) and (15) it follows that, for some finite $t_{a}$,

17 $$\begin{aligned} N ( t ) &\le e^{ - b_{2}t}N_{0} + \int _{0}^{t_{a}} e^{ - b_{2} ( t - \tau )} \bigl\vert b_{1} - \alpha I_{h} ( \tau ) \bigr\vert \,d\tau + \int _{t_{a}}^{t} e^{ - b_{2} ( t - \tau )} \bigl( b_{1} - \alpha I_{h} ( \tau ) \bigr) \,d\tau \\ &\le e^{ - b_{2}t}N_{0} + C ( t_{a} ) + \int _{t_{a}}^{t} e^{ - b_{2} ( t - \tau )} \bigl( b_{1} - \alpha I_{h} ( \tau ) \bigr) \,d\tau \\ &\le N_{0} + C ( t_{a} ) + \frac{1 - e^{ - b_{2} ( t - t_{a} )}}{b_{2}}\sup _{t_{a} \le t < + \infty } \bigl\vert b_{1} - \alpha I_{h} ( t ) \bigr\vert \\ &\le N_{0} + C ( t_{a} ) + b_{2}^{ - 1} \sup_{t_{a} \le t < + \infty } \bigl\vert b_{1} - \alpha I_{h} ( t ) \bigr\vert < + \infty,\quad \forall t \in \mathbf{R}_{0 +}, \end{aligned}$$

and property (ii) is proved. Since by Theorem 1 all the subpopulations are nonnegative all the time, property (ii) implies that they are also bounded all the time, and property (iii) is proved. □

### Remark 2

Note that Theorems 1–2 hold irrespectively of the vaccination and treatment control laws $V ( t ) = k_{V} ( t )S ( t )$ and $T ( t ) = k_{T} ( t )I_{h} ( t )$ of gains $k_{V}, k_{T}:\mathbf{R}_{0 +} \to \mathbf{R}_{0 +} $, which also covers the absence of one of both such controls. In particular, note from (15) that the total population is not constant through time in general because of the recruitment and disease mortality in its differential form (14). This fact is also clearly viewable in some simulated experiments of Sect. 4.

## Equilibrium points and stability results

In this section, we discuss the equilibrium points and their associated properties of local and global stability. As a final combined result of the local stability with the global stability and the nonnegativity properties proved in the former section, we establish the global asymptotic stability.

### Disease-free equilibrium point and its local stability and instability properties

The following result is concerned with the disease-free equilibrium point and its local stability properties if the basic reproduction number is less than one and the control gains converge to constant values. The result visualizes the dependence of the basic reproduction number with the asymptotic values of the control gains. Basically, the reproduction number is seen to become smaller as the limit control gains increase as time tends to infinity. In other words, the stability of the disease-free equilibrium point is improved by the vaccination and treatment control compared to the control-free situation. In parallel, we see that the reachable susceptible and recovered disease-free equilibrium values can be monitored by appropriate choices of the limit control gains.

#### Theorem 3

*Assume that* $\beta ( t ) \to \beta _{0}$, $k_{V} ( t ) \to k_{V0}$, *and* $k_{T} ( t ) \to k_{T0}$ *as* $t \to \infty $. *Then the following properties hold*:

(i) *There is a unique disease*-*free equilibrium point*
  18 $$ x_{df}^{*}: = \lim_{t \to \infty } x ( t ) = \bigl( S_{df}^{*}, E_{df}^{*}, I_{sdf}^{*}, I_{hdf}^{*}, A_{df}^{*}, R_{df}^{*} \bigr) ^{T} = \bigl( S_{df}^{*}, 0, 0, 0, 0, R_{df}^{*} \bigr) ^{T}, $$
  *where*
  19 $$\begin{aligned} &S_{df}^{*} = \frac{b_{1} + \eta R_{df}^{*}}{b_{2} + \eta + k_{V0}} = \frac{b_{1} ( b_{2} + \eta )}{b_{2} ( b_{2} + \eta + k_{V0} )}, \end{aligned}$$
  20 $$\begin{aligned} &R_{df}^{*} = \frac{k_{V0}S_{df}^{*}}{b_{2} + \eta } = \frac{k_{V0}b_{1}}{b_{2} ( b_{2} + \eta + k_{V0} )}, \end{aligned}$$
  *leading to a total population at the disease*-*free equilibrium point*:
  21 $$ N_{df}^{*} = S_{df}^{*} + R_{df}^{*} = \frac{b_{1}}{b_{2}}. $$
(ii) *Suppose*, *in addition*, *that* $k_{V0} = 0$, *that is*, *there is no limiting vaccination control*. *Then the basic reproduction number is*
  22 $$ R_{0} = \frac{\beta _{0}\gamma b_{1}}{b_{2} ( b_{2} + \gamma )} \biggl( \frac{ p_{s}}{ b_{2} + \tau _{0}} + \frac{ \beta _{hr}p_{h}}{b_{2} + \alpha + \tau _{0} + k_{T0}} + \frac{\beta _{ar} p_{a}}{ b_{2} + \tau _{0}} \biggr). $$
  *If this number is less than one*, *then the disease*-*free equilibrium point is locally asymptotically stable in the sense of Lyapunov*. *If it exceeds one*, *then the disease*-*free equilibrium point is unstable*.
(iii) *Assume that* $k_{V0} \ne 0$. *Then the basic reproduction number is*
  23 $$ R_{0} = \frac{\beta _{0}\gamma b_{1} ( b_{2} + \eta )}{b_{2} ( b_{2} + \gamma ) ( b_{2} + \eta + k_{V0} )} \biggl( \frac{ p_{s}}{ b_{2} + \tau _{0}} + \frac{ \beta _{hr}p_{h}}{b_{2} + \alpha + \tau _{0} + k_{T0}} + \frac{\beta _{ar} p_{a}}{ b_{2} + \tau _{0}} \biggr). $$
  *If this number is less than one*, *then the disease*-*free equilibrium point is locally asymptotically stable in the sense of Lyapunov*. *If it exceeds one*, *then the disease*-*free equilibrium point is unstable*.

#### Proof

Property (i) follows directly by equalizing to zero (1)–(6) with $E_{df}^{*} = I_{sdf}^{*} = I_{hdf}^{*} = A_{df}^{*} = 0$ and the constraints $\beta ( t ) \to \beta _{0}$, $k_{V} ( t ) \to k_{V0}$, and $k_{T} ( t ) \to k_{T0}$ as $t \to \infty $. This leads directly to (19)–(20), which summed up yield (21). To prove property (ii), first note that the Jacobian matrix of the linearized trajectory solution of (1)–(6) is

24 $$ \mathbf{A}_{df}^{*} = \begin{bmatrix} - ( b_{2} + k_{V0} ) & 0 & - \beta _{0} S_{df}^{*} & - \beta _{0}\beta _{hr}S_{df}^{*} & - \beta _{0}\beta _{ar}S_{df}^{*} & \eta \\ 0 & - ( b_{2} + \gamma ) & \beta _{0}S_{df}^{*} & \beta _{0}\beta _{hr}S_{df}^{*} & \beta _{0}\beta _{ar}S_{df}^{*} & 0 \\ 0 & \gamma p_{s} & - ( b_{2} + \tau _{0} ) & 0 & 0 & 0 \\ 0 & \gamma p_{h} & 0 & - ( b_{2} +\alpha + \tau _{0} + k_{T0} ) & 0 & 0 \\ 0 & \gamma p_{a} & 0 & 0 & - ( b_{2} + \tau _{0} ) & 0 \\ k_{V0} & 0 & \tau _{0} & \tau _{0} + k_{T0} & \tau _{0} & - ( b_{2} + \eta ) \end{bmatrix}. $$

If there is no limit vaccination control, then, in particular, it becomes

25 $$\begin{aligned} \mathbf{A}_{df0}^{*}& = \mathbf{A}_{df}^{*} ]_{ k_{V0} = 0} \\ & = \begin{bmatrix} - b_{2} & 0 & - \beta _{0} S_{df}^{*} & - \beta _{0}\beta _{hr}S_{df}^{*} & - \beta _{0}\beta _{ar}S_{df}^{*} & \eta \\ 0 & - ( b_{2} + \gamma ) & \beta _{0}S_{df}^{*} & \beta _{0}\beta _{hr}S_{df}^{*} & \beta _{0}\beta _{ar}S_{df}^{*} & 0 \\ 0 & \gamma p_{s} & - ( b_{2} + \tau _{0} ) & 0 & 0 & 0 \\ 0 & \gamma p_{h} & 0 & - ( b_{2} + \alpha + \tau _{0} + k_{T0} ) & 0 & 0 \\ 0 & \gamma p_{a} & 0 & 0 & - ( b_{2} + \tau _{0} ) & 0 \\ 0 & 0 & \tau _{0} & \tau _{0} + k_{T0} & \tau _{0} & - ( b_{2} + \eta ) \end{bmatrix}, \end{aligned}$$

so that it has two stable eigenvalues $- b_{2} < 0$ and $- ( b_{2} + \eta ) < 0$, and thus $\mathbf{A}_{df0}^{*}$ is a stability matrix if and only if the following fourth-order matrix of is also a stability matrix:

26 $$ \bar{\mathbf{A}}_{df0}^{*} = \begin{bmatrix} - ( b_{2} + \gamma ) & \beta _{0}S_{df}^{*} & \beta _{0}\beta _{hr}S_{df}^{*} & \beta _{0}\beta _{ar}S_{df}^{*} \\ \gamma p_{s} & - ( b_{2} + \tau _{0} ) & 0 & 0 \\ \gamma p_{h} & 0 & - ( b_{2} + \alpha + \tau _{0} + k_{T0} ) & 0 \\ \gamma p_{a} & 0 & 0 & - ( b_{2} + \tau _{0} ) \end{bmatrix} = \mathbf{Q} + \mathbf{P}, $$

where **Q** is the transition matrix, and **P** is the transmission matrix, which are defined by

27 $$ \mathbf{Q} = \begin{bmatrix} - ( b_{2} + \gamma ) & 0 & 0 & 0 \\ \gamma p_{s} & - ( b_{2} + \tau _{0} ) & 0 & 0 \\ \gamma p_{h} & 0 & - ( b_{2} + \alpha + \tau _{0} + k_{T0} ) & 0 \\ \gamma p_{a} & 0 & 0 & - ( b_{2} + \tau _{0} ) \end{bmatrix} $$

and

28 $$ \mathbf{P} = \beta _{0}S_{df}^{*} \mathbf{P}_{0};\qquad \mathbf{P}_{0} = \begin{bmatrix} 0 & 1 & \beta _{hr} & \beta _{ar} \\ 0 & 0 & 0 & 0 \\ 0 & 0 & 0 & 0 \\ 0 & 0 & 0 & 0 \end{bmatrix}. $$

Note that **Q** is a lower-triangular stability matrix (thus nonsingular with inverse $\mathbf{Q}^{ - 1} = ( \mathbf{Q} _{ij}^{ - 1} )$). Then

29 $$ \bar{\mathbf{A}}_{df0}^{*} = \mathbf{Q} \bigl( \mathbf{I}_{4} + \mathbf{Q}^{ - 1}\mathbf{P} \bigr) = \bigl( \mathbf{I}_{4} + \beta _{0}S_{df}^{*} \mathbf{P}_{0}\mathbf{Q}^{ - 1} \bigr) \mathbf{Q} = \mathbf{Q} \bigl( \mathbf{I}_{4} + \beta _{0}S_{df}^{*} \mathbf{Q}^{ - 1}\mathbf{P}_{0} \bigr) $$

is a stability matrix if and only if the spectral radius $r ( \mathbf{PQ}^{ - 1} )$ of $\mathbf{PQ}^{ - 1}$ is less than one. Under the stronger condition that for any matrix norm, $\Vert \mathbf{PQ}^{ - 1} \Vert < 1$, since $r ( \mathbf{PQ}^{ - 1} ) \le \Vert \mathbf{PQ}^{ - 1} \Vert $, by the Banach perturbation lemma [54] we get:

30 $$ \bigl\Vert \bar{\mathbf{A}}_{df0}^{* - 1} \bigr\Vert \le \bigl\Vert \bigl( \mathbf{I}_{4} + \mathbf{Q}^{ - 1} \mathbf{P} \bigr)^{ - 1} \bigr\Vert \bigl\Vert \mathbf{Q}^{ - 1} \bigr\Vert \le \frac{ \Vert \mathbf{Q}^{ - 1} \Vert }{1 - \beta _{0}S_{df}^{*}r ( \mathbf{Q}^{ - 1}\mathbf{P}_{0} )} \le \frac{ \Vert \mathbf{Q}^{ - 1} \Vert }{1 - \Vert \mathbf{Q}^{ - 1}\mathbf{P} \Vert }, $$

so that $\Vert \bar{\mathbf{A}}_{df0}^{* - 1} \Vert $ if $\beta _{0}S_{df}^{*} < 1/r ( \mathbf{Q}^{ - 1}\mathbf{P}_{0} )$, which proves the sufficiency part. To prove the necessity part, note that:

(a) If $\beta _{0} = 0$, then $\bar{\mathbf{A}}_{df0}^{*} = \mathbf{I}_{4}$ is nonsingular;

(b) the eigenvalues of any matrix are continuous functions with respect to any of its entries;

(c) the only possibly nonunity eigenvalue of $\mathbf{I}_{4} + \mathbf{Q}^{ - 1}\mathbf{P}$ is $\lambda = 1 + \beta _{0}S_{df}^{*} ( \mathbf{Q} _{21}^{ - 1} + \beta _{hr}\mathbf{Q} _{31}^{ - 1} + \beta _{ar}\mathbf{Q} _{41}^{ - 1} )$, which is nonzero only if $\beta _{0}S_{df}^{*} < 1/r ( \mathbf{Q}^{ - 1}\mathbf{P}_{0} )$, which proves the “only if” part, where

31 $$ \mathbf{Q}^{ - 1} = \begin{bmatrix} - ( b_{2} + \gamma )^{ - 1} & 0 & 0 & 0 \\ - \frac{\gamma p_{s}}{ ( b_{2} + \gamma ) ( b_{2} + \tau _{0} )} & - ( b_{2} + \tau _{0} )^{ - 1} & 0 & 0 \\ - \frac{\gamma p_{h}}{ ( b_{2} + \gamma )}{ ( b_{2} + \alpha + \tau _{0} + k_{T0} )} & 0 & - ( b_{2} + \alpha + \tau _{0} + k_{T0} )^{ - 1} & 0 \\ - \frac{\gamma p_{a}}{ ( b_{2} + \gamma ) ( b_{2} + \tau _{0} )} & 0 & 0 & - ( b_{2} + \tau _{0} )^{ - 1} \end{bmatrix}, $$

so that the unique nonzero row of $\mathbf{Q}^{ - 1}\mathbf{P}$ is its first row such that

32 $$ \bigl( \mathbf{Q}^{ - 1}\mathbf{P}_{0} \bigr)_{11} = \mathbf{Q} _{21}^{ - 1} + \beta _{hr}\mathbf{Q} _{31}^{ - 1} + \beta _{ar}\mathbf{Q} _{41}^{ - 1}, $$

and thus

33 $$ \lambda = 1 - \frac{\beta _{0}\gamma S_{df}^{*}}{b_{2} + \gamma } \biggl( \frac{ p_{s}}{ b_{2} + \tau _{0}} + \frac{ \beta _{hr}p_{h}}{b_{2} + \alpha + \tau _{0} + k_{T0}} + \frac{\beta _{ar} p_{a}}{ b_{2} + \tau _{0}} \biggr) > 0 $$

with $S_{df}^{*} = \frac{b_{1}}{b_{2}}$ in the absence of vaccination from (19), and $\mathbf{I}_{4} + \mathbf{Q}^{ - 1}\mathbf{P}$ is nonsingular if and only if

34 $$ \frac{\beta _{0}\gamma b_{1}}{b_{2} ( b_{2} + \gamma )} \biggl( \frac{ p_{s}}{ b_{2} + \tau _{0}} + \frac{ \beta _{hr}p_{h}}{b_{2} + \alpha + \tau _{0} + k_{T0}} + \frac{\beta _{ar} p_{a}}{ b_{2} + \tau _{0}} \biggr) < 1, $$

where $p_{a} = 1 - p_{s} - p_{h}$. Since all the eigenvalues of the Jacobian matrix $\mathbf{A}_{df0}^{*}$ around the disease-free equilibrium point are in the stability region, the disease-free equilibrium point is locally asymptotically stable. If the basic reproduction number exceeds one, then the disease-free equilibrium point is unstable. Property (ii) is proved.

To prove property (iii), note that $A_{df}^{*}$ has the same determinant as

35 $$\begin{aligned} \hat{\mathbf{A}}_{df}^{*} ={}& \left [\textstyle\begin{array}{cccccc} - ( b_{2} + k_{V0} ) & 0 & - \beta _{0} S_{df}^{*} \\ 0 & - ( b_{2} + \gamma ) & \beta _{0}S_{df}^{*} \\ 0 & \gamma p_{s} & - ( b_{2} + \tau _{0} ) \\ 0 & \gamma p_{h} & 0 \\ 0 & \gamma p_{a} & 0 \\ 0 & 0 & \tau _{0} + \beta _{0} S_{df}^{*}\frac{k_{V0}}{b_{2} + k_{V0}} \end{array}\displaystyle \right . \\ &\left .\textstyle\begin{array}{cccccc} - \beta _{0}\beta _{hr}S_{df}^{*} & - \beta _{0}\beta _{ar}S_{df}^{*} & \eta \\ \beta _{0}\beta _{hr}S_{df}^{*} & \beta _{0}\beta _{ar}S_{df}^{*} & 0 \\ 0 & 0 & 0 \\ - ( b_{2} + \alpha + \tau _{0} + k_{T0} ) & 0 & 0 \\ 0 & - ( b_{2} + \tau _{0} ) & 0 \\ \tau _{0} + k_{T0} + \beta _{0} \beta _{hr}S_{df}^{*}\frac{k_{V0}}{b_{2} + k_{V0}} & \tau _{0} + \beta _{0} \beta _{ar}S_{df}^{*}\frac{k_{V0}}{b_{2} + k_{V0}} & - ( b_{2} + \eta ( 1 + \frac{k_{V0}}{b_{2} + k_{V0}} ) ) \end{array}\displaystyle \right ] \end{aligned}$$

with the last row defined by adding to it the first row multiplied by $\theta = - \frac{k_{V0}}{b_{2} + k_{V0}}$. The matrix $A_{df}^{*}$ defined in (24) is a stability matrix, so that its six eigenvalues are in the complex open left-hand-side plane, and $\hat{A}_{df}^{*}$ has two negative real eigenvalues $- ( b_{2} + k_{V0} ) < 0$ and $- ( b_{2} + \eta ( 1 + \frac{k_{V0}}{b_{2} + k_{V0}} ) ) < 0$ by direct inspection of (35). So, the product of the four remaining eigenvalues must be a positive amount in order that both determinants be equal and $A_{df}^{*}$ be a stability matrix. But by construction the remaining eigenvalues are those of the $4 \times 4$ submatrix $\bar{A}_{df0}^{*}$ of (26) being common to $A_{df}^{*}$ and $\hat{A}_{df}^{*}$ obtained by deleting from both the first and sixth rows and columns. It has been proved that such a submatrix is a stability matrix if and only if $R_{0}$ defined in (23) is less than one. As a result, $\hat{A}_{df}^{*}$ is a stability matrix if and only if $\bar{\mathbf{A}}_{df0}^{*}$ is a stability matrix. Then the rest of the proof is identical to that of property (ii). Then the local asymptotic stability of the disease-free equilibrium point holds if and only if (33) holds with $S_{df}^{*} = \frac{b_{1} ( b_{2} + \eta )}{b_{2} ( b_{2} + \eta + k_{V0} )}$ modified by the vaccination control from (19) related to its value $b_{1}/b_{2}$ in the vaccination-free case of property (ii). This results in the following condition:

36 $$ \frac{\beta _{0}\gamma b_{1} ( b_{2} + \eta )}{b_{2} ( b_{2} + \gamma ) ( b_{2} + \eta + k_{V0} )} \biggl( \frac{ p_{s}}{ b_{2} + \tau _{0}} + \frac{ \beta _{hr}p_{h}}{b_{2} + \alpha + \tau _{0} + k_{T0}} + \frac{\beta _{ar} p_{a}}{ b_{2} + \tau _{0}} \biggr) < 1, $$

and property (iii) is proved. □

#### Remark 3

Note that it is possible to quantify the attenuation of the basic reproduction number depending on the limiting control gains related to the control-free situation or related to the case where only the vaccination or the treatment control is used. This quantification of the improvement of the reproduction number by the control action (in the sense that it is reduced) becomes explicit by comparing (23) and its particular cases resulting when one or both controls are zero, that is, with its particular case given by (22). This concern is an important issue associated with the use of controls against the control-free case since the reproduction number is mathematically related to the relative stability of the Jacobian matrix around the disease-free equilibrium point being a measure of how far its dominant eigenvalue is from the unstable region, which is the closed complex right-hand side plane. Biologically, the basic reproduction number is interpreted as the average of primary contagions caused by each infectious individual. This number should be less than one to asymptotically remove the infection. Simple calculations of comparisons of (23) with (22) lead to

$$\begin{aligned} &R_{0} ( k_{V0}, k_{T0} ) = C_{aVT} ( k_{V0}, k_{T0} )R_{0} ( 0,0 ), \\ &R_{0} ( k_{V0}, 0 ) = C_{aV} ( k_{V0} )R_{0} ( 0,0 ), \\ &R_{0} ( 0, k_{T0} ) = C_{aT} ( k_{T0} )R_{0} ( 0,0 ), \end{aligned}$$

and

$$\begin{aligned} &C_{aVT} ( k_{V0}, k_{T0} ) = \frac{ ( b_{2} + \eta ) ( b_{2} + \alpha + \tau _{0} )}{ ( b_{2} + \eta + k_{V0} ) ( b_{2} + \alpha + \tau _{0} + k_{T0} )} \\ &\phantom{C_{aVT} ( k_{V0}, k_{T0} ) =}{}\times \frac{ ( p_{s} + \beta _{ar} p_{a} ) ( b_{2} + \alpha + \tau _{0} + k_{T0} ) + \beta _{hr}p_{h} ( b_{2} + \tau _{0} )}{ ( p_{s} + \beta _{ar} p_{a} ) ( b_{2} + \alpha + \tau _{0} ) + \beta _{hr}p_{h} ( b_{2} + \tau _{0} )}, \\ &C_{aT} ( k_{T0} ) = C_{a} ( 0,k_{T0} ) = \frac{ ( b_{2} + \alpha + \tau _{0} ) ( p_{s} + \beta _{ar} p_{a} ) ( b_{2} + \alpha + \tau _{0} + k_{T0} ) + \beta _{hr}p_{h} ( b_{2} + \tau _{0} )}{ ( b_{2} + \alpha + \tau _{0} + k_{T0} ) ( p_{s} + \beta _{ar} p_{a} ) ( b_{2} + \alpha + \tau _{0} ) + \beta _{hr}p_{h} ( b_{2} + \tau _{0} )}, \\ &C_{aV} ( k_{V0} ) = C_{a} ( k_{V0}, 0 ) = \frac{b_{2} + \eta }{b_{2} + \eta + k_{V0}}, \end{aligned}$$

where $R_{0} ( k_{V0}, k_{T0} )$ is the reproduction number (23) denoted as a function of the two limiting control gains to facilitate the immediate discussion which follows, and, in particular, one of the two gains can be zero, and (22) is obtained if both of them are zero, which is the basic reproduction number of the control-free case, and the coefficients $C_{a (\cdot )} (\cdot,\cdot )$ are the corresponding attenuation coefficients of the basic reproduction number under one or both controls.

### Endemic equilibrium point and its attainability and local stability

The next result gives the existence and uniqueness of the endemic equilibrium point and it gives conditions for its attainability, nonattainability in the sense that all its components are nonnegative, and the case where not all of them are nonnegative. Note that it is understood that the attainability (or reachability) of the endemic equilibrium point does not mean, in principle, that it is stable, but that it is feasible related to the positivity property of the SE(Is)(Ih)AR model in the sense that the state trajectory solution has nonnegative components all the time under any nonnegative initial conditions (Theorem 1). The local stability conditions of the endemic equilibrium point are also given related to the basic reproduction number value exceeding unity. Under weak supplementary conditions on the parameters and the limit values of the transmission rate and control gains, the endemic equilibrium point is both attainable and locally asymptotically stable if the disease-free one is unstable so if the reproduction number exceeds unity.

#### Theorem 4

*Assume that*

$$ \beta ( t ) \to \beta _{e}, \qquad k_{V} ( t ) \to k_{Ve} \quad\textit{and}\quad k_{T} ( t ) \to k_{Te}\quad \textit{as } t \to \infty $$

*and*, *correspondingly to a basic reproduction number equal to unity*, *define the critical transmission rate*

37 $$ \beta _{c} = \frac{b_{2} ( b_{2} + \gamma ) ( b_{2} + \eta + k_{V0} )}{\beta _{0}\gamma b_{1} ( b_{2} + \eta )}\frac{ ( b_{2} + \tau _{0} ) ( b_{2} + \alpha + \tau _{0} + k_{T0} )}{ ( p_{s} + \beta _{ar} p_{a} ) ( b_{2} + \alpha + \tau _{0} + k_{T0} ) + \beta _{hr}p_{h} ( b_{2} + \tau _{0} )}. $$

*Then the following properties hold*:

(i) *If* $\beta _{e} \ge \beta _{c}$, *then there is a unique endemic equilibrium point* $x_{\mathrm{end}}^{*}: = \lim_{t \to \infty } x ( t ) = ( S_{\mathrm{end}}^{*}, E_{\mathrm{end}}^{*}, I_{\mathrm{send}}^{*}, I_{\mathrm{hend}}^{*}, A_{\mathrm{end}}^{*}, R_{\mathrm{end}}^{*} ) ^{T}$, *with all positive components*, *that is*, *it is attainable*.
(ii) *Assume that* $\beta _{e} = \beta _{0} = \beta _{c}$ (*that is*, $R_{0} = 1$), $k_{Ve} = k_{V0}$, *and* $k_{Te} = k_{T0}$. *Then* $S_{\mathrm{end}}^{*} = S_{df}^{*}$. *In addition*, $E_{\mathrm{end}}^{*} \le 0$, $I_{\mathrm{send}}^{*} \le 0$, $I_{\mathrm{hend}}^{*} \le 0$, *and* $A_{\mathrm{end}}^{*} \le 0$ *if* $\beta _{e} = \beta _{0} \le \beta _{c}$ (*that is*, *if* $R_{0} \le 1$), *provided that* $b_{1} - b_{2} \le a = \frac{k_{V0}\eta ( b_{1} - \tau _{0} )}{b_{2} ( \eta + \tau _{0} ) + b_{1}\tau _{0}\eta } $, *or*, *in particular*, *provided that* $\tau _{0} \le b_{1} \le b_{2}$, *or if* $b_{1} \le b_{2}$ *and* $k_{Ve} = 0$. *As a result*, *the infective endemic equilibrium subpopulations are negative if* $b_{1} - b_{2} < a$ *and* $R_{0} < 1$ *or if* $b_{1} - b_{2} \le a$ *and* $R_{0} < 1$, *so that the endemic equilibrium point is not attainable*.
(iii) *If* $\beta _{e} = \beta _{0} > \beta _{c}$ (*that is*, $R_{0} > 1$), *then the attainable endemic equilibrium point is locally asymptotically stable in the sense of Lyapunov*.

#### Proof

Firstly, equalize to zero (1)–(6) for nonzero equilibrium values of infective subpopulations $E_{\mathrm{end}}^{*}, I_{\mathrm{send}}^{*}, I_{\mathrm{hend}}^{*}$, and $A_{\mathrm{end}}^{*}$ to get the components of the endemic equilibrium point. We get:

38 $$\begin{aligned} &S_{\mathrm{end}}^{*} = \frac{b_{1} + \eta R_{\mathrm{end}}^{*}}{b_{2} + \beta _{e} ( I_{\mathrm{send}}^{*} + \beta _{hr}I_{\mathrm{hend}}^{*} + \beta _{ar}A_{\mathrm{end}}^{*} ) + k_{Ve}}, \end{aligned}$$

39 $$\begin{aligned} &E_{\mathrm{end}}^{*} = \frac{ ( \beta I_{\mathrm{send}}^{*} + \beta _{hr} I_{\mathrm{hend}}^{*} + \beta _{ar}A_{\mathrm{end}}^{*} ) S_{\mathrm{end}}^{*}}{b_{2} + \gamma }, \end{aligned}$$

40 $$\begin{aligned} &A_{\mathrm{end}}^{*} = C_{A}E_{\mathrm{end}}^{*} = \frac{\gamma p_{a}}{b_{2} + \tau _{0} } E_{\mathrm{end}}^{*}, \end{aligned}$$

41 $$\begin{aligned} &I_{\mathrm{send}}^{*} = C_{Is} E_{\mathrm{end}}^{*} = \frac{\gamma p_{s}}{b_{2} + \tau _{0} } E_{\mathrm{end}}^{*}, \end{aligned}$$

42 $$\begin{aligned} &I_{\mathrm{hend}}^{*} = C_{Ih}E_{\mathrm{end}}^{*} = \frac{\gamma p_{h}}{b_{2} + \alpha + \tau _{0} + k_{Te}}E_{\mathrm{end}}^{*}, \end{aligned}$$

43 $$\begin{aligned} &R_{\mathrm{end}}^{*} = \frac{\tau _{0} ( I_{\mathrm{send}}^{*} + A_{\mathrm{end}}^{*} ) + ( k_{Te} + \tau _{0} ) I_{\mathrm{hend}}^{*} + k_{Ve}S_{\mathrm{end}}^{*}}{b_{2} + \eta }, \end{aligned}$$

which yields that all the infective subpopulations (40)–(42) are linked to the endemic one (39) through positive real constants $C_{A} = \frac{\gamma p_{a}}{b_{2} + \tau _{0}}$, $C_{Is} = \frac{\gamma p_{s}}{b_{2} + \tau _{0}}$, and $C_{Ih} = \frac{\gamma p_{h}}{b_{2} + \alpha + \tau _{0} + k_{Te}}$. Then substituting (40)–(42) into (39), we get

44 $$ E_{\mathrm{end}}^{*} = \frac{\gamma }{b_{2} + \gamma } S_{\mathrm{end}}^{*} E_{\mathrm{end}}^{*}\beta _{e} \biggl( \frac{p_{s}}{b_{2} + \tau _{0} } + \frac{\beta _{hr}p_{h}}{b_{2} + \alpha + \tau _{0} + k_{Te}} + \frac{\beta _{ar} p_{a}}{b_{2} + \tau _{0} } \biggr). $$

Since $E_{\mathrm{end}}^{*} \ne 0$ at the endemic equilibrium point, we obtain

45 $$\begin{aligned} S_{\mathrm{end}}^{*}& = \frac{b_{2} + \gamma }{\gamma \beta _{e}}\frac{ ( b_{2} + \alpha + \tau _{0} + k_{Te} ) ( b_{2} + \tau _{0} )}{ ( p_{s} + \beta _{ar}p_{a} ) ( b_{2} + \alpha + \tau _{0} + k_{Te} ) + \beta _{hr}p_{h} ( b_{2} + \tau _{0} )} \end{aligned}$$

46 $$\begin{aligned} &= \frac{b_{2} + \gamma }{\gamma \beta _{e}}\frac{ \frac{\gamma p_{h}}{C_{Ih}} + \frac{\gamma p_{s}}{C_{Is}} }{\frac{ ( p_{s} + \beta _{ar} p_{a} ) \gamma p_{h}}{C_{Ih}} + \frac{\beta _{hr}p_{h}\gamma p_{s}}{C_{Is}}} \\ &= \frac{b_{2} + \gamma }{\gamma \beta _{e}}\frac{ p_{h} C_{Is} + p_{s}C_{Ih} }{ ( p_{s} + \beta _{ar} p_{a} ) p_{h}C_{Is} + \beta _{hr}p_{h}p_{s}C_{Ih}}. \end{aligned}$$

Now by replacing (40)–(42) into (43) and comparing the resulting constraint with (38), we get

47 $$\begin{aligned} &E_{\mathrm{end}}^{*} \biggl( \frac{\eta }{b_{2} + \tau _{0}} \bigl[ \tau _{0} ( C_{Is} + C_{a} ) + ( k_{Te} + \tau _{0} ) C_{Ih} - \beta _{e} ( C_{Is} + \beta _{hr}C_{Ih} + \beta _{ar}C_{a} ) \bigr] \\ &\qquad{} - \beta _{e} ( C_{Is} + \beta _{hr}C_{Ih} + \beta _{ar}C_{a} ) S_{\mathrm{end}}^{*} \biggr) \\ &\quad = \biggl( b_{2} + K_{Ve} \biggl( 1 - \frac{\eta }{b_{2} + \tau _{0}} \biggr) \biggr) S_{\mathrm{end}}^{*} - b_{1}, \end{aligned}$$

so that

48 $$\begin{aligned} E_{\mathrm{end}}^{*} &= \frac{ ( b_{2} ( b_{2} + \tau _{0} ) + K_{Ve} ( b_{2} + \tau _{0} - \eta ) )S_{\mathrm{end}}^{*} - b_{1} ( b_{2} + \tau _{0} )}{ \eta ( \tau _{0} ( C_{Is} + C_{a} ) + ( k_{Te} + \tau _{0} ) C_{Ih} ) - ( C_{Is} + \beta _{hr}C_{Ih} + \beta _{ar}C_{a} ) ( \beta _{e} \eta + \beta _{e} ( b_{2} + \tau _{0} )S_{\mathrm{end}}^{*} )} \end{aligned}$$

49 $$\begin{aligned} &= \frac{\Delta _{1} - \Delta _{2}}{\Delta _{3} - \Delta _{4}}, \end{aligned}$$

where

50 $$\begin{aligned} &\Delta _{1} = \bigl( b_{2} ( b_{2} + \tau _{0} ) + K_{Ve} ( b_{2} + \tau _{0} - \eta ) \bigr) ( p_{h} C_{Is} + p_{s}C_{Ih} ) ( b_{2} + \gamma ), \end{aligned}$$

51 $$\begin{aligned} &\Delta _{2} = \gamma \beta _{e}b_{1} ( b_{2} + \tau _{0} ) \bigl( ( p_{s} + \beta _{ar} p_{a} ) p_{h}C_{Is} + \beta _{hr}p_{h}p_{s}C_{Ih} \bigr), \end{aligned}$$

52 $$\begin{aligned} &\Delta _{3} = \bigl( \gamma \beta _{e}\eta \bigl( \tau _{0} ( C_{Is} + C_{a} ) + ( k_{Te} + \tau _{0} ) C_{Ih} \bigr) \bigr) \bigl( ( p_{s} + \beta _{ar} p_{a} ) p_{h}C_{Is} + \beta _{hr}p_{h}p_{s}C_{Ih} \bigr), \end{aligned}$$

53 $$\begin{aligned} &\Delta _{4} = ( C_{Is} + \beta _{hr}C_{Ih} + \beta _{ar}C_{a} ) \bigl( \gamma \beta _{e}^{2} \eta \bigl( ( p_{s} + \beta _{ar} p_{a} ) p_{h}C_{Is} + \beta _{hr}p_{h}p_{s}C_{Ih} \bigr) \\ &\phantom{\Delta _{4} =}{}+ ( b_{2} + \tau _{0} ) \beta _{e} ( b_{2} + \gamma ) ( p_{h} C_{Is} + p_{s}C_{Ih} ) \bigr). \end{aligned}$$

Note the following facts:

*Fact 1*: $S_{\mathrm{end}}^{*}$ is positive by (44) and is unique.

*Fact 2*: For sufficiently large transmission rate $\beta _{e} \ge \beta _{c}$ and some critical value of the transmission rate $\beta _{c} > 0$, $\Delta _{2} > \Delta _{1}$, and $\Delta _{4} > \Delta _{3}$, $E_{\mathrm{end}}^{*}$ is positive and unique by (47)–(48).

*Fact 3*: By Facts 1–2 and (37)–(42) the endemic equilibrium point $x_{\mathrm{end}}^{*}: = \lim_{t \to \infty } x ( t ) = ( S_{\mathrm{end}}^{*} , E_{\mathrm{end}}^{*}, I_{\mathrm{send}}^{*}, I_{\mathrm{hend}}^{*}, A_{\mathrm{end}}^{*}, R_{\mathrm{end}}^{*} ) ^{T}$ is unique with all positive components (so that it is attainable) for a sufficiently large transmission rate.

*Fact 4*: If $\beta _{e} = \beta _{0}$, $k_{Ve} = k_{V0}$, and $k_{Te} = k_{T0}$, then the basic reproduction number (23) exceeds unity; equivalently, if $\beta _{c} = \beta _{e} = \beta _{0}$ in (37), then by (44)–(45) and (40)–(43) all the endemic equilibrium subpopulations are nonnegative if $R_{0} \ge 1$ and $b_{1} - b_{2} \ge a = \frac{k_{V0}\eta ( b_{1} - \tau _{0} )}{b_{2} ( \eta + \tau _{0} ) + b_{1}\tau _{0}\eta } $. In the same way, $E_{\mathrm{end}}^{*} \le 0$, $I_{\mathrm{send}}^{*} \le 0$, $I_{\mathrm{hend}}^{*} \le 0$, and $A_{\mathrm{end}}^{*} \le 0$ if $b_{1} - b_{2} \le a$, or, in particular, if $\tau _{0} \le b_{1} \le b_{2}$, or if $b_{1} \le b_{2}$ and $k_{Ve} = 0$. As a result, the infective endemic equilibrium subpopulations are negative if $b_{1} - b_{2} < a$ and $R_{0} \le 1$ or if $b_{1} - b_{2} \le a$ and $R_{0} < 1$, so that the endemic equilibrium point is not attainable.

As a result, property (i) follows from Fact 1. On the other hand, property (ii) follows from property (i) Facts 1–4, since from (23) and (45) we conclude that if $\beta _{e}$ is defined by (37) and $\beta _{e} = \beta _{0}$, $k_{Ve} = k_{V0}$, and $k_{Te} = k_{T0}$, then $R_{0} = 1$ and $S_{\mathrm{end}}^{*} = S_{df}^{*}$. The remaining conditions on the nonattainability of the equilibrium point of property (ii) follow from (45) and (48) and the proportionality relations of the infectious subpopulations to the exposed one in (40)–(42).

To prove property (iii), note that the linearized trajectory solution around the endemic equilibrium is defined by the Jacobian matrix

54 $$\begin{aligned} \mathbf{A}_{\mathrm{end}}^{*} = {}& \left [\textstyle\begin{array}{cccccc} - ( b_{2} + \beta _{e} ( I_{\mathrm{send}}^{*} + \beta _{hr}I_{\mathrm{hend}}^{*} + \beta _{ar}A_{\mathrm{end}}^{*} ) + K_{Ve} ) & 0 & - \beta _{e} S_{\mathrm{end}}^{*} \\ \beta _{e} ( I_{\mathrm{send}}^{*} + \beta _{hr}I_{\mathrm{hend}}^{*} + \beta _{ar}A_{\mathrm{end}}^{*} ) & - ( b_{2} + \gamma ) & \beta _{e} S_{\mathrm{end}}^{*} \\ 0 & \gamma p_{s} & - ( b_{2} + \tau _{0} ) \\ 0 & \gamma p_{h} & 0 \\ 0 & \gamma p_{a} & 0 \\ K_{Ve} & 0 & \tau _{0} \end{array}\displaystyle \right ., \\ &\left .\textstyle\begin{array}{cccccc} - \beta _{e}\beta _{hr}S_{\mathrm{end}}^{*} & - \beta _{e}\beta _{ar}S_{\mathrm{end}}^{*} & \eta \\ \beta _{e}\beta _{hr}S_{\mathrm{end}}^{*} & \beta _{e}\beta _{ar}S_{\mathrm{end}}^{*} & 0 \\ 0 & 0 & 0 \\ - ( b_{2} + \alpha + \tau _{0} + k_{Te} ) & 0 & 0 \\ 0 & - ( b_{2} + \tau _{0} ) & 0 \\ \tau _{0} + k_{Te} & \tau _{0} & - ( b_{2} + \eta ) \end{array}\displaystyle \right ], \end{aligned}$$

which has the same determinant as the matrix

55 $$\begin{aligned} \hat{\mathbf{A}}_{\mathrm{end}}^{*} ={}& \left [\textstyle\begin{array}{cccccc} - ( b_{2} + \beta_{e} ( I_{\mathrm{send}}^{*} + \beta_{hr}I_{\mathrm{hend}}^{*} + \beta_{ar}A_{\mathrm{end}}^{*} ) + K_{Ve} ) & 0 & - \beta_{e} S_{\mathrm{end}}^{*} \\ \beta_{e} ( I_{\mathrm{send}}^{*} + \beta_{hr}I_{\mathrm{hend}}^{*} + \beta_{ar}A_{\mathrm{end}}^{*} ) & - ( b_{2} + \gamma ) & \beta_{e} S_{\mathrm{end}}^{*} \\ 0 & \gamma p_{s} & - ( b_{2} + \tau_{0} ) \\ 0 & \gamma p_{h} & 0 \\ 0 & \gamma p_{a} & 0 \\ 0 & 0 & \tau_{0} + \vert \theta_{e} \vert \beta_{e} S_{\mathrm{end}}^{*} \end{array}\displaystyle \right . \\ &\left .\textstyle\begin{array}{cccccc} - \beta_{e}\beta_{hr}S_{\mathrm{end}}^{*} & - \beta_{e}\beta_{ar}S_{\mathrm{end}}^{*} & \eta \\ \beta_{e}\beta_{hr}S_{\mathrm{end}}^{*} & \beta_{e}\beta_{ar}S_{\mathrm{end}}^{*} & 0 \\ 0 & 0 & 0 \\ - ( b_{2} + \alpha + \tau_{0} + k_{Te} ) & 0 & 0 \\ 0 & - ( b_{2} + \tau_{0} ) & 0 \\ \tau_{0} + k_{Te} + \vert \theta_{e} \vert \beta_{e}\beta_{hr} S_{\mathrm{end}}^{*} & \tau_{0} + \vert \theta_{e} \vert \beta_{e}\beta_{ar} S_{\mathrm{end}}^{*} & - ( b_{2} + \eta ) ( 1 + \vert \theta_{3} \vert ) \end{array}\displaystyle \right ] \end{aligned}$$

since the last row is defined by adding to it the first row multiplied by $\theta _{e} = - \frac{k_{Ve}}{b_{2} + \beta _{e} ( I_{\mathrm{send}}^{*} + \beta _{hr}I_{\mathrm{hend}}^{*} + \beta _{ar}A_{\mathrm{end}}^{*} ) + K_{Ve}}$. Now invoke a close reasoning as that previously used for the disease-free Jacobian matrix (26) versus (35), which has the same determinant. As a result, we conclude that $\mathbf{A}_{\mathrm{end}}^{*}$ and $\hat{\mathbf{A}}_{\mathrm{end}}^{*}$ are nonsingular if and only if the $4 \times 4$ submatrix $\bar{\mathbf{A}}_{df0}^{*}$ defined in (26) is nonsingular. In particular, $\mathbf{A}_{\mathrm{end}}^{*}$ and $\hat{\mathbf{{A}}}_{\mathrm{end}}^{*}$ are stability matrices if and only if the $4 \times 4$ submatrix $\bar{\mathbf{A}}_{df0}^{*}$ defined in (26) is a stability matrix, and, equivalently, they are unstable if and only if the $4 \times 4$ submatrix $\bar{\mathbf{A}}_{df0}^{*}$ is unstable since two of the eigenvalues of both of them are always stable by construction. Then, under the constraint $\beta _{e} = \beta _{0} = \beta _{c}$, if follows that $\mathbf{A}_{\mathrm{end}}^{*}$ and $\hat{\mathbf{A}}_{\mathrm{end}}^{*}$ are nonsingular if and only if $\beta _{e}S_{\mathrm{end}}^{*} > 1/r ( \mathbf{Q}^{ - 1}\mathbf{P}_{0} )$. If such an inequality becomes an inequality, then either a stable or unstable eigenvalue becomes a critical eigenvalue so that $\hat{\mathbf{{A}}}_{\mathrm{end}}^{*}$ and $\mathbf{A}_{\mathrm{end}}^{*}$ become singular.

It is now proved that for $R_{0} > 1$ (equivalently, if $\beta _{e} = \beta _{0} > \beta _{c}$), the endemic equilibrium point is locally asymptotically stable or, equivalently, the nonsingular matrix $\hat{\mathbf{{A}}}_{\mathrm{end}}^{*}$ (and, equivalently, the nonsingular matrix $\mathbf{A}_{\mathrm{end}}^{*}$) has all eigenvalues in the open complex left-hand side plane. Assume on the contrary that for $R_{0} > 1$, the endemic equilibrium point is unstable. Since the disease-free one is unstable too [Theorem 3(iii)], a stable limit cycle has to surround the endemic equilibrium point, since according to the Poincaré–Bendixson theorem:

- If no stable limit cycle exists, then no attractor exists, and the SE(Is)(Ih)AR model is not globally Lyapunov stable, which contradicts Theorem 2(iii). So, a stable limit cycle should exist.
- The stable limit cycle has to surround one of the equilibrium points only since all the singular values surrounding it have a net Poincaré index equal to unity, and the Poincaré index of two singular points would be −2 if both are saddle points, +2 if no one is a saddle point, and 0 if one is a saddle point while the other is not.
- The limit cycle cannot surround the disease-fee equilibrium point since then any solution trajectory violates the nonnegativity property (Theorem 1).

However, if the endemic equilibrium point is unstable and is surrounded by a stable limit cycle, then the Jacobian matrix $\mathbf{A}_{\mathrm{end}}^{*}$ of the linearized solution trajectory around the endemic equilibrium point within a small neighborhood centered at it has to have a critically stable eigenvalue, but this implies that the inequality constraint $\beta _{e}S_{\mathrm{end}}^{*} > 1/r ( \mathbf{Q}^{ - 1}\mathbf{P}_{0} )$ becomes violated by an equality implying that the reproduction number is unity. Therefore it is impossible that for $R_{0} > 1$ (with the disease-free equilibrium point then being unstable), the endemic equilibrium point is also unstable and surrounded by a stable limit cycle. Therefore if $R_{0} > 1$, then the disease-free equilibrium point is unstable, and the endemic one is locally asymptotically stable. Property (iii) is proved. □

### Global asymptotic stability

Some conclusions can be obtained about global stability from the characterizations of the equilibrium points or periodic solutions and their local stability properties. Note from Theorem 2 that the SE(Is)(Ih)AR model is globally stable under nonnegative initial finite values of all subpopulations. It is proved that there is a unique disease-free equilibrium point and a unique endemic one. On the other hand, the critical transmission rate $\beta _{c}$ was defined by equalizing the limit transmission rate (23) to unity. We saw that if the current limit transmission rate equalizes to $\beta _{0}$ and it is smaller than its critical value $\beta _{c}$, then the endemic equilibrium point is not attainable, whereas the disease-free one was proved to be asymptotically stable in Theorem 2. Also, if the current limit transmission rate exceeds the critical value, then the disease-free equilibrium point is unstable, whereas the endemic one is locally asymptotically stable. As a result, only one of the equilibrium points is locally asymptotically depending on the value of the limit transmission rate compared to its critical value, which gives a unity basic reproduction number. On the other hand, we saw in the last part of the proof of Theorem 4 that no limit cycle can exist surrounding any of the two equilibrium points or both of them.

#### Theorem 5

*Assume that*

1. $\beta ( t ) \to \beta _{0} = \beta _{e}$, $k_{V} ( t ) \to k_{V0} = k_{Ve}$, *and* $k_{T} ( t ) \to k_{T0} = k_{Te}$ *as* $t \to \infty$,
2. $b_{1} - b_{2} < a = \frac{k_{V0}\eta ( b_{1} - \tau _{0} )}{b_{2} ( \eta + \tau _{0} ) + b_{1}\tau _{0}\eta }$.

Then the following properties hold:

1. The whole nonlinear linear SE(Is)(Ih)AR is globally asymptotically stable in the sense of Lyapunov with the disease-free equilibrium point being the only global attractor if $\beta _{0} = \beta _{e} < \beta _{c}$.
2. The whole nonlinear linear SE(Is)(Ih)AR is globally asymptotically stable in the sense of Lyapunov with the endemic equilibrium point being the only global attractor if $\beta _{0} = \beta _{e} > \beta _{c}$.

#### Outline of Proof

The proof is direct from the following previously proved results:

- Theorem 1 on the nonnegativity of any solution under nonnegative finite initial conditions,
- Theorem 2 on the global stability in the sense of Lyapunov of any nonnegative solution trajectory of the whole nonlinear SE(Is)(Ih)AR model,
- Theorem 3 on the local asymptotic stability in the sense of Lyapunov of the disease-free equilibrium point if $\beta _{0} < \beta _{c}$,
- Theorem 4 on the local asymptotic stability of the endemic equilibrium point in the sense of Lyapunov if $\beta _{0} = \beta _{e} > \beta _{c}$ with the additional results: (1) it is unique, (2) its attainability holds under the instability of the disease-free equilibrium point, and (3) no limit cycle can surround one or both equilibrium points.

 □

## Numerical worked examples

This section contains some numerical simulation examples illustrating the theoretical results introduced in Sects. 2 and 3. Therefore we consider the parameter values corresponding to COVID-19 and supplied in the background literature. Note that the estimation of model parameters from available data faces two main challenges: (i) the first one is the treatment of raw data. Data related to Covid-19 usually exhibit inconsistency and are subject to large uncertainties. Thus an exhaustive work of data preprocessing and analysis is needed before using them in parameter estimation procedures. (ii) Furthermore, the model has a large number of parameters making the estimation procedure complex. These facts make the estimation problem hard to be tackled, requiring a special attention as a focused topic. Since the paper is devoted to the mathematical properties of the model and to the effect of applying vaccination and antiviral control, we have employed the typical parameter values considered previously in the medical literature instead of starting from the parameter identification process. There is a broad consensus in the scientific community about the values considered for some parameters of the model, such as the basic reproduction numbers or average incubation periods. Therefore we believe that the presented results are representative of the possibilities and usefulness of the method.

The numerical value of the reproduction number has been obtained through Eq. (23) by using the numerical data collected in Table 1 from previous existing background literature. Previous works report different reproduction numbers depending on the place and moment of the outbreak since the social habits and lifestyle, interchange level of population with neighboring areas, and population density are eventually different these values are even different for the first and second waves since they are also strongly dependent on the intervention measures and rules. However, the situation described in the paper represents a benchmark to show the usefulness of the proposed approach.

**Table 1:** Parameter values employed in simulations

Parameter	Interpretation	Value	Source
$b_{{1}}$	Recruitment rate	57,554 years^−1^	[55], year 2018
$b_{{2}}$	Natural average death rate	1/85 years^−1^	[55]
*β*	Transmission rate of symptomatic	1/*N(0)*	[38], adjusted to provide a basic reproduction number between 5-6
$\beta _{ar}$	Specific transmission rate factor of asymptomatic	1	[56, 57]
$\beta _{hr}$	Specific transmission rate factor of severe cases (hospitalized)	1/50 (nominal)	Sensitivity analysis for $\beta _{hr} \in [ 1/10,1/100 ]$
*γ*	Average incubation period	1/5.5 days^−1^	[36]
*η*	Average immunity loss rate	0	[36, 56]
*α*	Mortality rate for severe cases associated with disease	12%	[58]
$\tau _{0}$	Average immune response rate	1/10 days^−1^	[36]
$p_{s}$	Fraction of slight cases	55%	[58, 59]
$p_{h}$	Fraction of severe cases	20%	[58, 59]

It has to be highlighted that reported data regarding COVID-19 exhibit high variability among outbreaks or are even inconsistent. Thus the parameter values could be subject to changes as further knowledge on the infection is attained. Moreover, parameters may suffer changes in time due to different public health policies implemented to fight against the spread of COVID-19. The simulations are performed with the initially estimated values given in Tables 1 and 2 for the specific demographic case of the Madrid Region (Comunidad de Madrid).

**Table 2:** Initial conditions for simulations

Population	Value
*S*(0)	6,778,382
*E*(0)	1
$I_{s}{(0)}$	0
$I_{h}{(0)}$	0
*A*(0)	0
*R*(0)	0
*N*(0)	6,778,383

**Figure 1:**
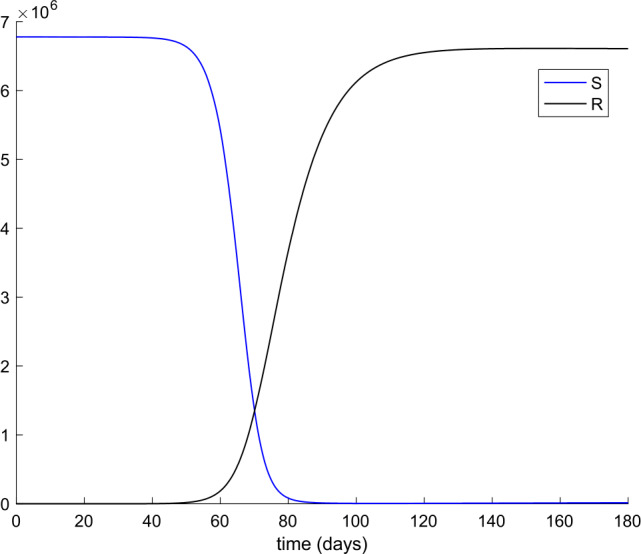
Evolution of susceptible and recovered in the absence of control actions

**Figure 2:**
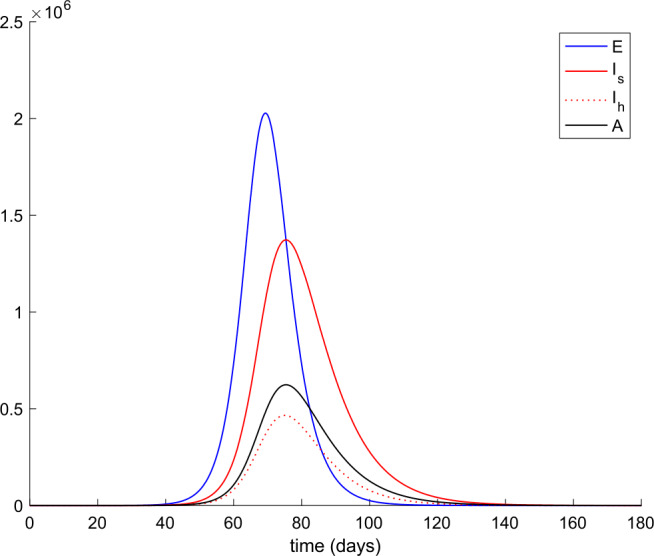
Evolution of exposed and infectious (slight, hospitalized, and asymptomatic) in the absence of control actions

From Table 2 we can deduce that all simulations start with the total population being susceptible and a single exposed case. Figures 1 and 2 display the evolution of all populations in the absence of control actions (vaccination and treatment). We concluded from Fig. 1 that the spreading of the disease would end up affecting the total population if no control action was taken, as it was concluded in [56] as well.

**Figure 3:**
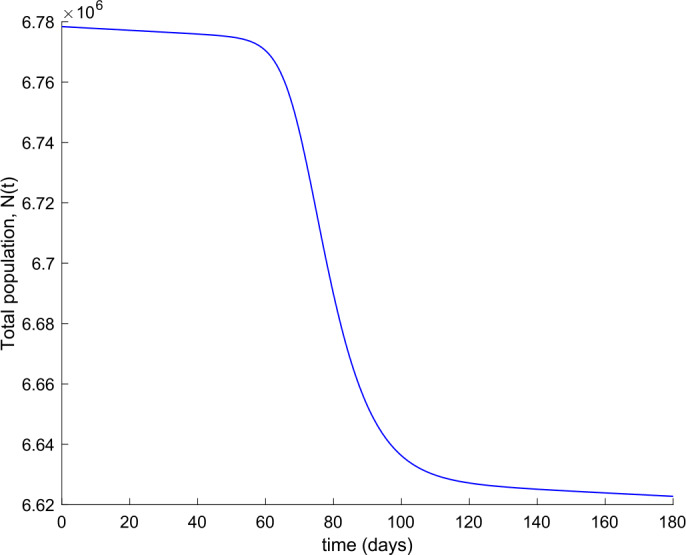
Evolution of the total population in the absence of external actions

**Figure 4:**
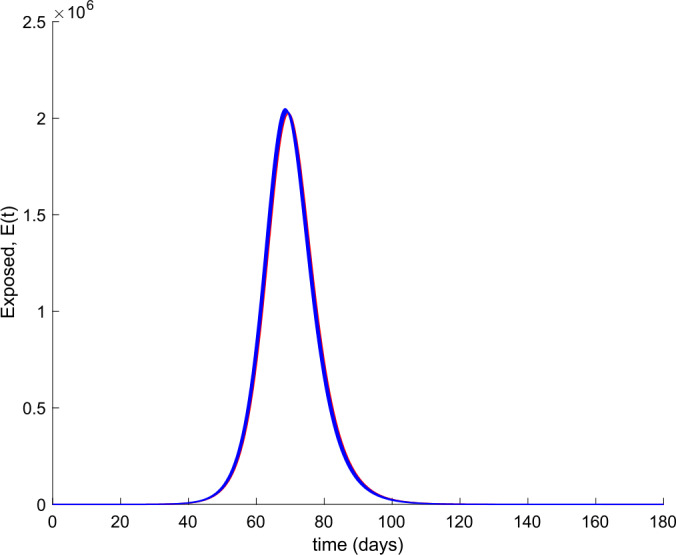
Variation of the number of exposed individuals with $\beta _{hr}$ in the absence of control actions

**Figure 5:**
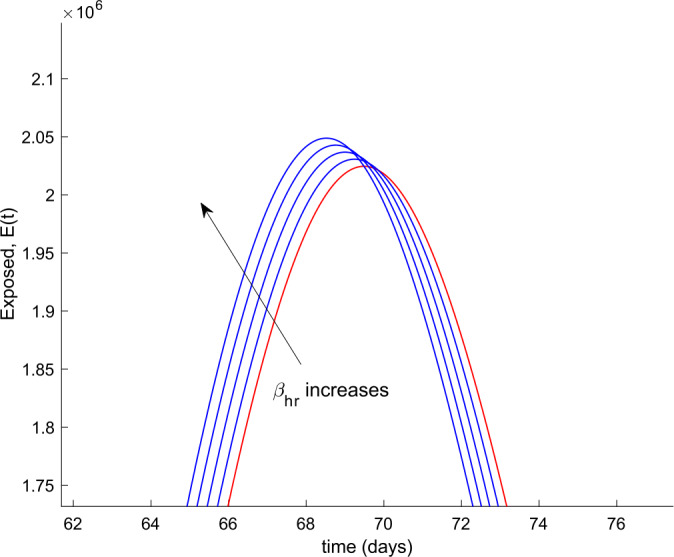
Zoom on the variation of the number of exposed individuals with $\beta _{hr}$ in the absence of control actions

We also observe in Fig. 2 a large number of severe infected people (hospitalized) attained at the peak. Such a large number of severe cases would definitely overflow the hospital available resources. To avoid this situation, two control actions, vaccination and treatment, are considered and analyzed in the following. Figure 3 displays the evolution of the total population, representing essentially the deaths caused by the disease. Note that the total population is not constant through time as theoretically discussed in Remark 2. Figures 4 and 5 show the results of the sensitivity analysis performed for $\beta _{hr}$ with values ranging between $\beta _{hr} =$1/10 and $\beta _{hr} =$1/100. We deduced that the shape of exposed does not change significantly as $\beta _{hr}$ changes. In addition, observe in Figs. 1 and 2 that the model solution is nonnegative and bounded as Theorems 1 and 2 establish.

**Figure 6:**
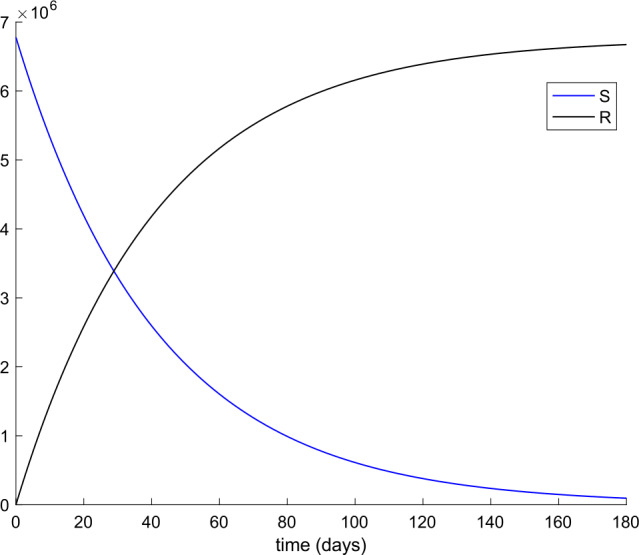
Evolution of susceptible and recovered when vaccination is applied and no treatment is used. The vaccination gain is set to $k_{V} = 0.001$

**Figure 7:**
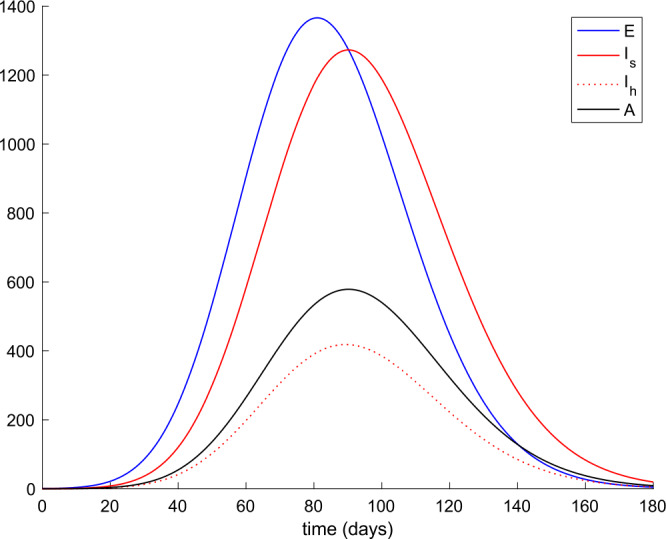
Evolution of exposed and infectious (slight, hospitalized, and asymptomatic) when vaccination is applied and no treatment is used. The vaccination gain is set to $k_{V} = 0.001$

**Figure 8:**
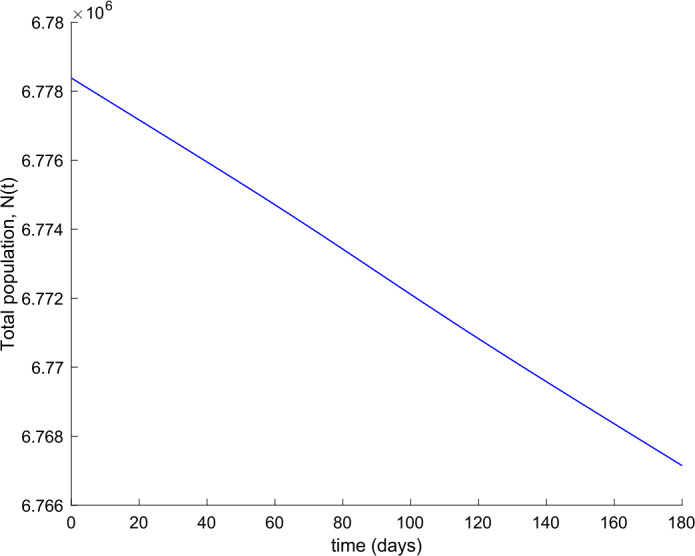
Evolution of the total population when vaccination is applied and no treatment is used. The vaccination gain is set to $k_{V} = 0.001$

Now we will discuss the effect of vaccination and treatment through simulation examples. Initially, we apply a vaccination action of $k_{V} = 0.001$ to the model while no treatment is used. In this case, we obtain Figs. 6, 7, and 8.

**Figure 9:**
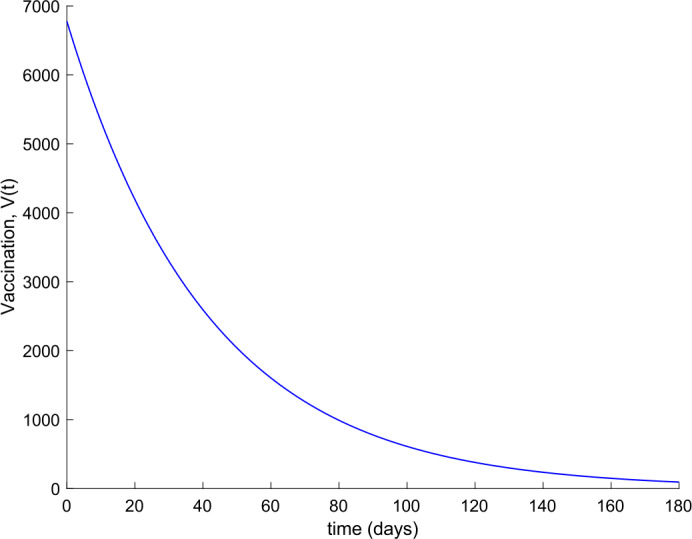
Vaccination action when $k_{V} = 0.001$ and no treatment is applied

**Figure 10:**
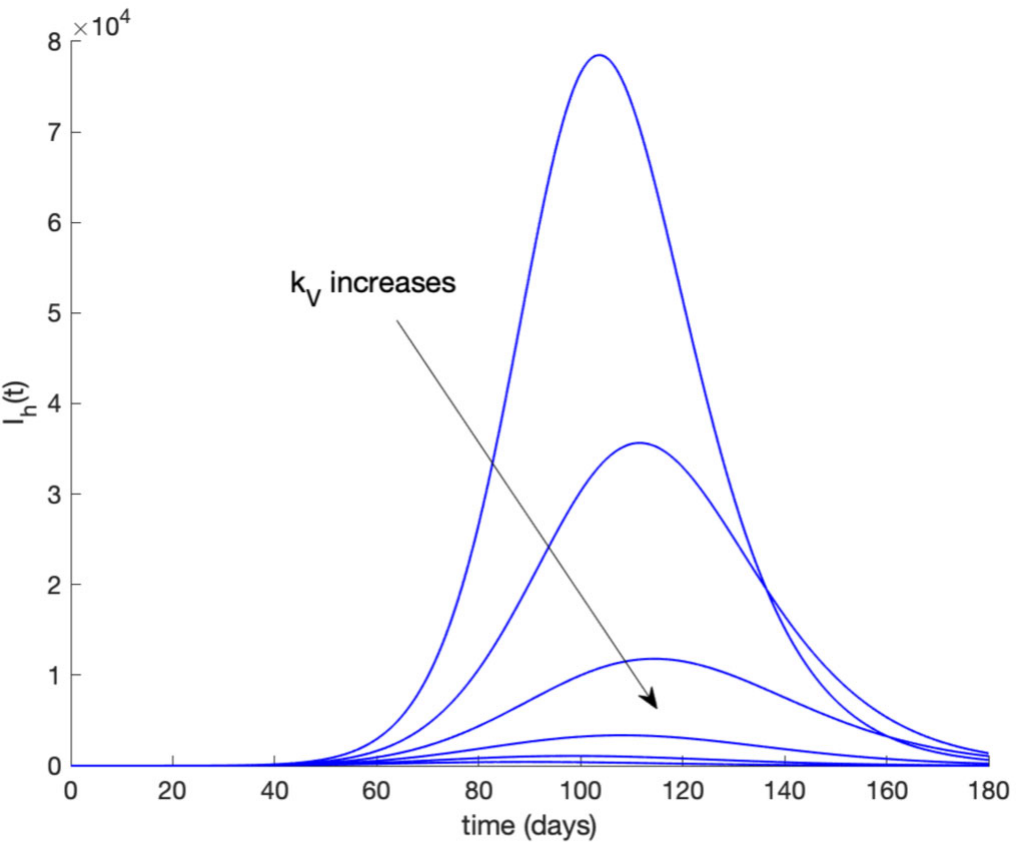
Effect of vaccination gain $k_{V}$ on the number of hospitalized infectious. The vaccination gain varies between $k_{V} = 0.0005$ and $k_{V} = 0.001$. No treatment control is applied

Observe in Fig. 7 a substantial reduction in the number of hospitalized cases, whereas Fig. 6 shows that the population becomes immune faster than in the absence of control actions, as it could be intuitively expected. Furthermore, the death toll is also reduced as can be concluded by comparing Figs. 3 and 8 regarding the evolution of the total population. Figure 9 displays the vaccination action needed. The great improvement in the disease incidence is achieved at the expense of a high effort in vaccination. Moreover, Fig. 10 shows the effect of changing the vaccination gain $k_{V}$ between $k_{V} =0.0005$ and $k_{V} =0.001$ on the evolution of hospitalized individuals. As the gain increases, the number of hospitalized cases declines. As it is claimed in Sect. 3, the use of vaccination improves the behavior of the coronavirus spread.

**Figure 11:**
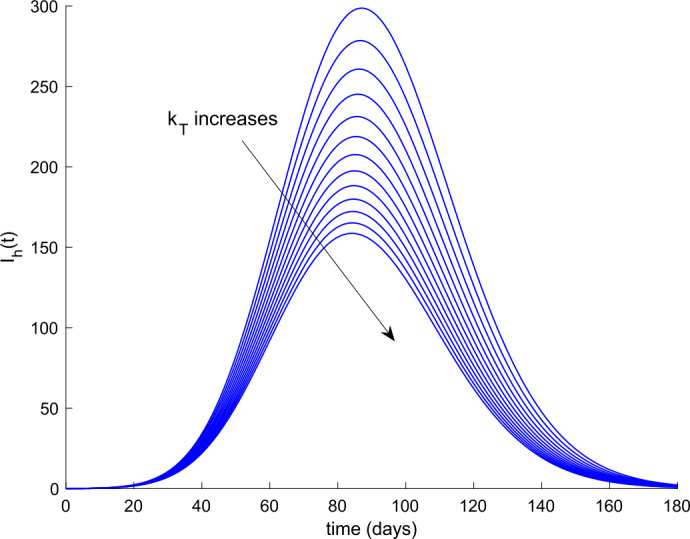
Effect of both actions, vaccination and treatment, on the evolution of hospitalized cases. The value of $k_{V}$ is fixed to $k_{V} = 0.001$, and the value of $k_{T}$ varies from $k_{T} = 0.002$ to $k_{T} = 0.008$

Now the value of $k_{V}$ is fixed to $k_{V} = 0.001$, and the value of $k_{T}$ ranges from $k_{T} =0.002$ to $k_{T} =0.008$. Figure 11 displays the evolution of hospitalized cases in this situation. We conclude that the combined application of treatment along with vaccination drastically reduces the number of severe cases and prevents the overflow of hospital resources.

**Figure 12:**
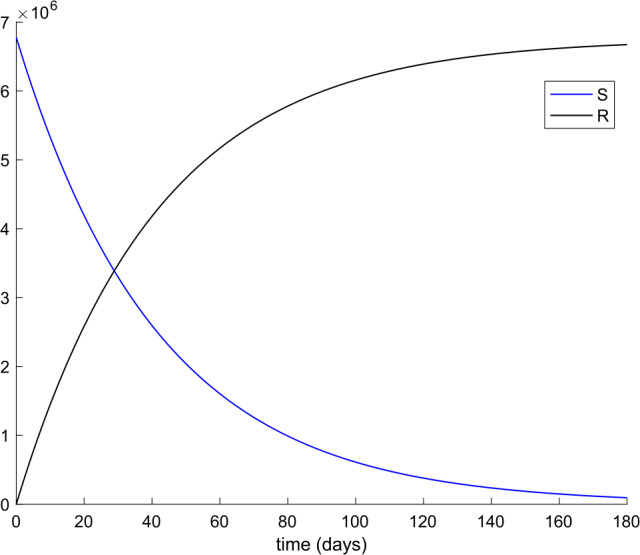
Evolution of Susceptible and Immune when vaccination and treatment are applied with $k_{V} = 0.001$ and $k_{T} = 0.004$

**Figure 13:**
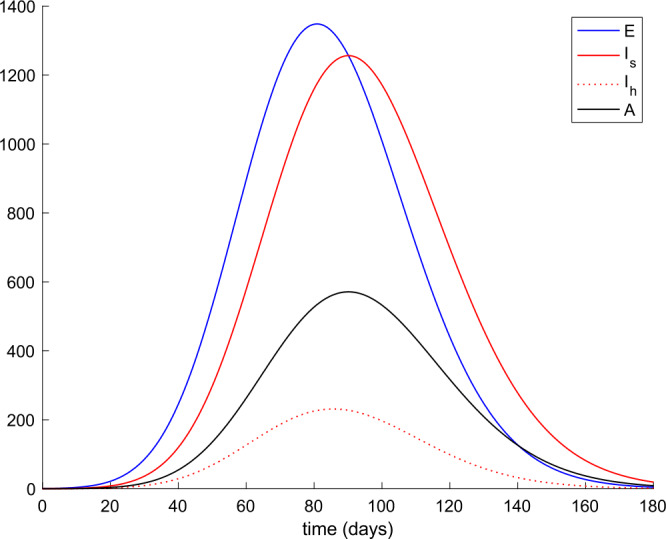
Evolution of exposed, infectious (slight and hospitalized), and asymptomatic when vaccination and treatment are applied with $k_{V} = 0.001$ and $k_{T} = 0.004$

**Figure 14:**
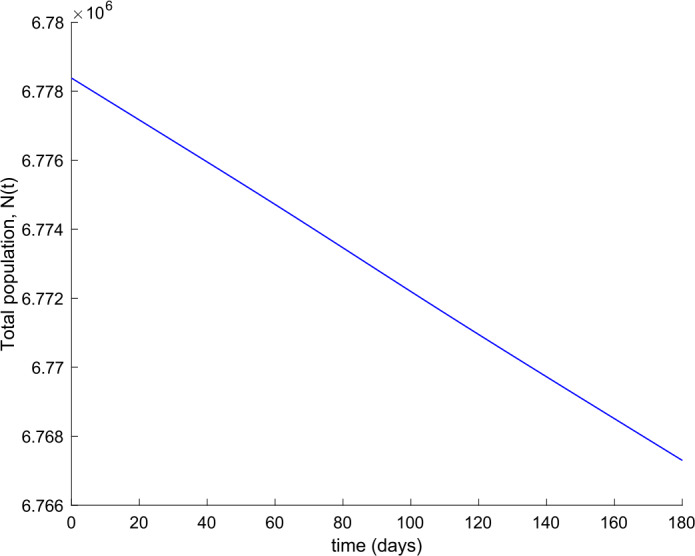
Evolution of the total population when vaccination and treatment are applied with $k_{V} = 0.001$ and $k_{T} = 0.004$

**Figure 15:**
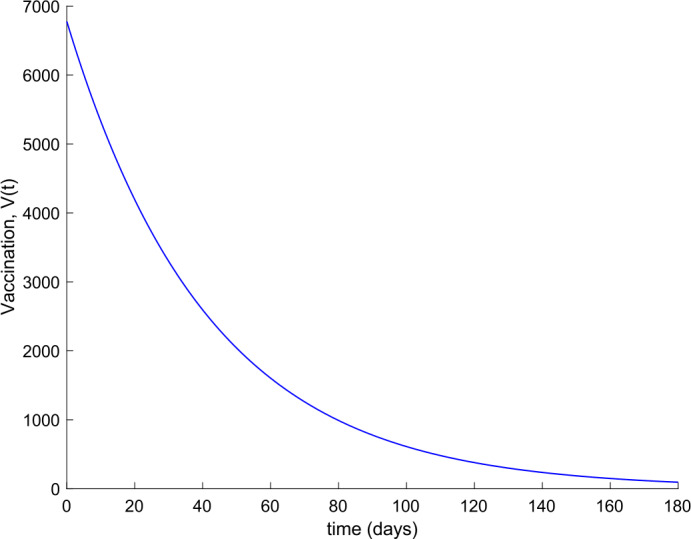
Vaccination action when $k_{V} = 0.001$ and $k_{T} = 0.004$

**Figure 16:**
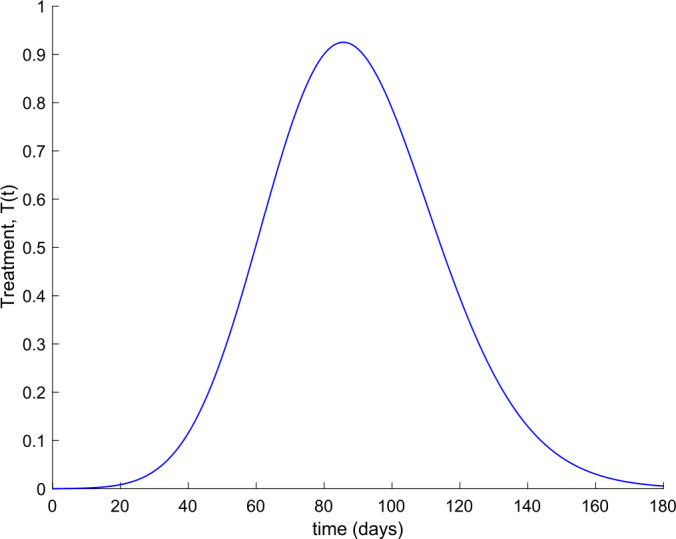
Treatment action when $k_{T} = 0.004$

Figures 11, 12, 13, and 14 display the evolution of all subpopulations, including the total one when, in particular, $k_{V} = 0.001$ and $k_{T} = 0.004$. The corresponding vaccination and treatment controls are displayed in Figs. 15 and 16, respectively.

**Figure 17:**
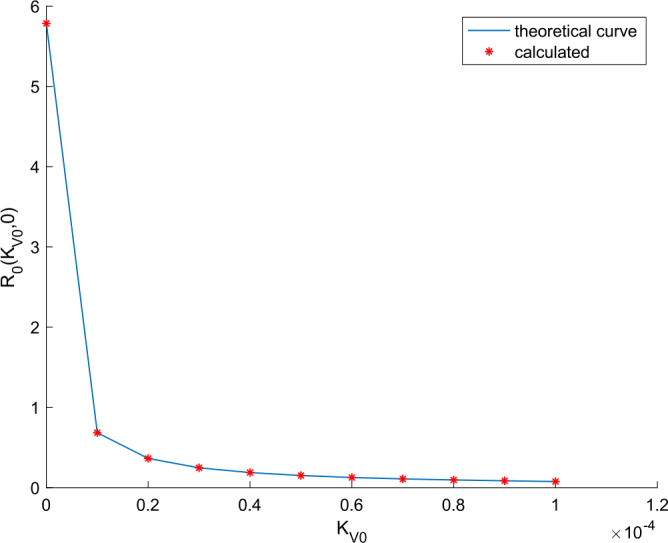
Variation of the basic reproduction number with vaccination and without treatment

**Figure 18:**
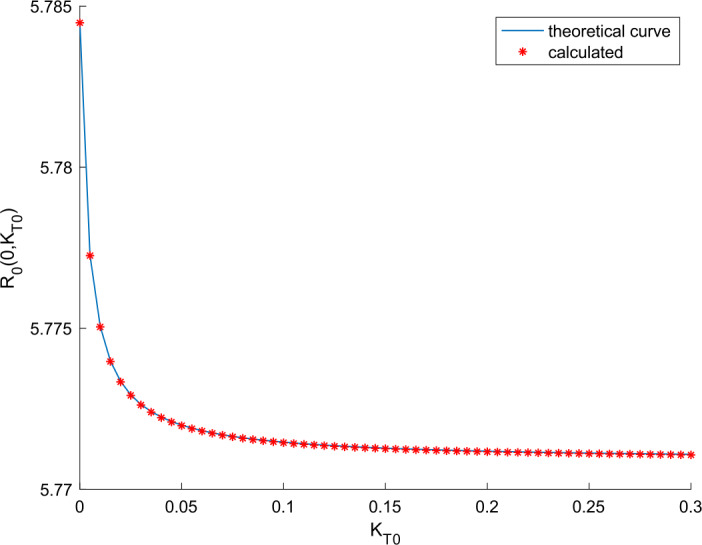
Variation of the basic reproduction number with treatment and without vaccination

Overall, vaccination and treatment have the effect of counteracting the effects of coronavirus COVID-19 spreading. The larger these actions, the higher the improvement at the expense of higher efforts and therefore a higher cost. With the proposed model, quantitative prediction of the improvement and action efforts can be done as shown with the simulation results. The basic reproduction number in the absence of external actions is calculated through (23) as $R(0,0) =$5.78. When $k_{T} = 0$, Remark 3 allows calculating the obtained reproduction number when a vaccination gain is applied. The shape of the curve is depicted in Fig. 17 along with the calculated values of the reproduction number for some particular vaccination gains. On the other hand, when there is no vaccination action and a treatment is applied, the basic reproduction number changes a depicted in Fig. 18. We deduce from Figs. 17 and 18 that vaccination has a stronger effect in modifying the reproduction number and controlling the epidemic spreading than the application of treatment. Thus vaccination is proposed as the main way for controller spreading, whereas treatment is devoted to heal the hospitalized cases and recover their heath the soonest and safest as possible. Furthermore, Remark 3 (or in an equivalent graphical way, Fig. 17) can be used as a guideline to calculate the critical vaccination gain that provides a unity reproduction number. Thus, if we make

$$ R_{0} ( k_{V0}, 0 ) = 1 = C_{aV} ( k_{V0} )R_{0} ( 0,0 ) = \frac{b_{2} + \eta }{b_{2} + \eta + k_{V0}}R(0,0), $$

then we can isolate $k_{V}(t) = k_{V0}$ as $k_{V0} = (b_{2} + \eta ) ( R(0,0) - 1 ) = 6.425 \cdot 10^{ - 6}$ for the parameters considered. If a vaccination gain larger than this critical value is used, then the reproduction number is less than unity. Consequently, the theoretical developments contained in Sect. 3 provide useful guidelines to design the vaccination action aimed at controlling COVID-19 spread.

### Remark 4

We observed the following features from both the theoretical analysis and the performed numerical experiments:

(1) The obtained results allow the calculation of the amounts of vaccination and treatment efforts (given by the vaccination and treatment gains) needed to counteract the spread of Covid-19 depending on the estimated original reproduction number R(0,0) in the absence of controls since this control-free reproduction number is related to and higher than the respective current reproduction numbers $R_{0} ( k_{V0}, 0 )$, $R_{0} ( 0, k_{T0} )$, and $R_{0} ( k_{V0}, k_{T0} )$ in the presence of one or both controls of respective limit gains $k_{V0}$ for the vaccination control and $k_{T0}$ for the treatment control. Since the reproduction number is proved to be dependent on the control gains and reduced related to its value in the control-free case, it turns out that it is easier to keep the illness under low incidence levels by the correct planning of the vaccination policies. Note that since the reproduction number reflects the number of infections derived at a first stage from each primary one, keeping such a number under unity is crucial to asymptotically extinguish the disease by leaving the disease-free equilibrium as the unique attainable global asymptotic attractor.

(2) For a given population, the control gains allow determining the number of vaccination and treatment doses needed to keep the pandemic under control. This information is crucial to go ahead with the purchase agreements with pharmaceutical companies with the aim of investing the optimal economical burden in fighting against the infection, especially, in a situation where state public finances are subject to a great stress. The general information is also useful for the sanitary authorities for planning their vaccination policies, including the managing and monitoring aspects of generation, administration, storage, and distribution of the vaccination and treatment doses. This featured point follows as a result of the proved mentioned dependence and reduction of the reproduction number on the control gains.

## Conclusions and potential related future research

This paper has developed an SE(Is)(Ih)AR epidemic model which involves six subpopulations and can be useful for modelling the COVId-19 pandemic. The infectious subpopulation of the standard SEIR model is split into three subpopulations, namely, the slight infectious individuals who do not need hospital care, the hospitalized ones who are seriously infected, and the asymptomatic ones. The three above infectious subpopulations are originated by different transitions from the exposed subpopulation. The proposed and discussed epidemic model is eventually assumed to be subject to vaccination and treatment controls. In general, the transmission rate and the feedback control gains can be monitored to be time-varying along the transients.

The properties of nonnegativity and boundedness of all the subpopulations are proved under any given finite nonnegative initial conditions. Also, the disease-free and the endemic equilibrium points are explicitly calculated, and their uniqueness and local asymptotic stability properties are also investigated with respect to the reference unity value of the basic reproduction number. It is shown that just one of them, depending on the value of the basic reproduction number, is the unique global asymptotic attractor. It is also proved that no limit cycle can surround any individual or jointly both equilibrium points if the transmission rate and the control gains converge asymptotically to constant values. Finally, some numerical examples are developed and discussed based on previously tested parameterizations of COVID-19 available in the background literature data.

We plan to focus the future investigation on the estimation of the disease transmission rate from recorded infection data while fixing the remaining disease modeling parameters from supplied tested medical background data and to integrate its estimation in the model running. A second idea for future investigation is designing the control strategies so that the maximum availability of beds for both ordinary hospitalization and intensive care unit management can be prefixed under certain upper-bounding constraints to keep some resources for its use in other sanitary needs. This concern seems to be important since now there is a very high pressure on hospital derived from CoVID pandemic making difficult the ordinary management of resources.

## Data Availability

The data supporting the results of the tested numerical results are included in the list of references and cited in the appropriate locations.
